# Minimally Invasive Versus open AbdominoThoracic Esophagectomy for esophageal carcinoma (MIVATE) — study protocol for a randomized controlled trial DRKS00016773

**DOI:** 10.1186/s13063-020-04966-z

**Published:** 2021-01-11

**Authors:** Felix Nickel, Pascal Probst, Alexander Studier-Fischer, Henrik Nienhüser, Jana Pauly, Karl-Friedrich Kowalewski, Sebastian Weiterer, Philipp Knebel, Markus K. Diener, Markus A. Weigand, Markus W. Büchler, Thomas Schmidt, Beat P. Müller-Stich

**Affiliations:** 1 Department of General, Visceral and Transplantation Surgery, Heidelberg University Hospital, Im Neuenheimer Feld 420, 69120 Heidelberg, Germany; 2 Department of Anaesthesiology, Heidelberg University Hospital, Im Neuenheimer Feld 420, 69120 Heidelberg, Germany

**Keywords:** Minimally invasive esophagectomy, Esophageal cancer, Ivor-Lewis esophagectomy, Linear stapled anastomosis, Circular stapled anastomosis, Randomized controlled trial, Comprehensive complication index, Fast track, Enhanced recovery after surgery, Expertise-based

## Abstract

**Background:**

The only curative treatment for most esophageal cancers is radical esophagectomy. Minimally invasive esophagectomy (MIE) aims to reduce postoperative morbidity, but is not yet widely established. Linear stapled anastomosis is a promising technique for MIE because it is quite feasible even without robotic assistance. The aim of the present study is to compare total MIE with linear stapled anastomosis to open esophagectomy (OE) with circular stapled anastomosis with special regard to postoperative morbidity in an expertise-based randomized controlled trial (RCT).

**Methods/design:**

This superiority RCT compares MIE with linear stapled anastomosis (intervention) to OE with circular stapled anastomosis (control) for Ivor-Lewis esophagectomy. It was initiated in February 2019, and recruitment is expected to last for 3 years. For inclusion, patients must be 18 years of age or more with a resectable primary malignancy in the distal esophagus. Participants with tumor localizations above the azygos vein, metastasis, or infiltration into adjacent tissue will be excluded. In an expertise-based approach, the allocated treatment will only be carried out by the single most experienced surgeon of the surgical center for each respective technique. The sample size was calculated with 20 participants per group for the primary endpoint postoperative morbidity according to comprehensive complication index (CCI) within 30 postoperative days. Secondary endpoints include anastomotic insufficiency, pulmonary complications, other intra- and postoperative outcome parameters such as estimated blood loss, operative time, length of stay, short-term oncologic endpoints, adherence to a standardized fast-track protocol, postoperative pain, and postoperative recovery (*QoR-15*). Quality of life (*SF-36*, *CAT EORTC QLQ-C30*, *CAT EORTC QLQ-OES18*) and oncological outcomes are evaluated with 60 months follow-up.

**Discussion:**

MIVATE is the first RCT to compare OE with circular stapled anastomosis to total MIE with linear stapled anastomosis exclusively for intrathoracic anastomosis. The expertise-based approach limits bias due to heterogeneity of surgical expertise. The use of a dedicated fast-track protocol in both OE and MIE will shed light on the role of the access strategy alone in this setting. The findings of this study will serve to define which approach has the best perioperative outcome for patients requiring esophagectomy.

**Trial registration:**

German Clinical Trials Register DRKS00016773. Registered on 18 February 2019.

**Supplementary Information:**

The online version contains supplementary material available at 10.1186/s13063-020-04966-z.

## Background

The only curative treatment for most esophageal cancers is esophagectomy with systematic radical lymphadenectomy, which often requires abdominothoracic surgery due to its anatomic position. The open Ivor-Lewis procedure, which is one of the most common approaches, classically consists of a median laparotomy and a right-lateral thoracotomy. Despite improved outcomes in high-volume centers over the last decades, the approach is still associated with postoperative overall complication rates as high as 40–80% and relevant mortality rates. Major parts of morbidity and mortality are caused by the occurrence of anastomotic insufficiency and pulmonary complications [1, 2].

Minimally invasive approaches and fast-track protocols attempt to lower postoperative complication rates [3, 4]. Consequently, there have been several studies attempting to lower postoperative morbidity by using minimally invasive approaches for esophagectomy [5–10]. The existing studies evaluating minimally invasive esophagectomy (MIE) have shown technical feasibility and suggest similar oncological outcomes [11]. The different surgical approaches relevant for esophagectomy can be differentiated according to surgical access, site of anastomosis, and anastomotic technique. In terms of surgical access, the procedures can be performed via open technique, hybrid, or total minimally invasive surgery comprising conventional laparoscopic/thoracoscopic as well as robotic approaches. The anastomoses can be performed with manual suturing or as stapled anastomoses with either end-to-side circular stapled anastomosis or side-to-side linear stapled anastomosis, the latter being a commonly used anastomotic technique in other fields such as obesity surgery [12]. The combination of these aspects results in a great variety of possible techniques for esophagectomy that need to be carefully evaluated.

MIE is gaining more popularity due to expected lower postoperative morbidity compared to open and hybrid procedures while oncological outcomes have been promising as well [13]. It is hypothesized that with the total minimally invasive approach, postoperative morbidity can be reduced while oncological outcomes stay at least equivalent to open surgery [14]. Studies that have evaluated MIE in a randomized setting so far mostly used either intrathoracic circular stapled end-to-side anastomosis or cervical anastomosis [7, 15]. A simple and reliable alternative to presented techniques is the totally minimally invasive approach with side-to-side linear stapled intrathoracic anastomosis that was established in obesity surgery. Especially for gastric bypass surgery, linear stapled side-to-side anastomosis is a well-established technique and has the potential to decrease the number of postoperative complications in esophagectomy [12]. Reasons advocating linear stapled anastomosis include technical feasibility of intrathoracic anastomosis, no requirement for robotic assistance, and the fact that it is well-established and safe in the frame of other surgical disciplines such as gastric bypass surgery for obesity.

However, until now, no randomized trials for total MIE with linear stapled side-to-side anastomosis vs. open esophagectomy (OE) with circular stapled end-to-side intrathoracic anastomosis exist. Besides the novel character of the study regarding its comparison of two surgical strategies that have not been compared so far, this trial also addresses a methodical problem that often accompanies surgical clinical trials, i.e., the different skillset and experience level of participating surgeons as one of the main sources for heterogeneity and bias [16–18]. Multicenter randomized controlled trials (RCTs) are the centerpieces in evidence-based surgery. These RCTs require that surgeons in different centers with varying training and expertise of a certain intervention are pooled in order to reach patient numbers that are sufficient for proper statistical analysis. For optimal outcomes, surgeons should only perform procedures that they are experienced and comfortable with. This is especially momentous when investigating complex procedures such as esophagectomy and can lead to poor recruitment and biased results. A possibility of coping with this issue are clinical trials that are performed in an expertise-based manner [19], in which the surgeons performing surgeries for trials exclusively are the most experienced and peer-acknowledged senior surgical members of staff. Since esophagectomy is a treatment prone to this type of bias, MIVATE is conducted in an expertise-based manner.

The aim of the present study is thus to compare postoperative morbidity between total MIE with linear stapled side-to-side anastomosis to open surgery with circular stapled end-to-side anastomosis in an expertise-based randomized trial.

## Methods/design

### Setting

This is a single-center RCT at the Department of General, Visceral and Transplantation Surgery, Heidelberg University Hospital, Germany. It was initiated in February 2019, and recruitment is expected to last for 3 years. The study protocol was accepted by the independent Ethics Committee of the University of Heidelberg (registration number S-317/2017) before start of the study. The trial was registered at DRKS under registration number DRKS00016773 on February 18, 2019 [20]. No Secondary Identifying Numbers such as a Universal Trial Number have been assigned. Recommendations of the SPIRIT (Standard protocol items: Recommendations for Interventional Trials) checklist (Additional file 1) were followed [21].

### Patient recruitment

Recruitment exclusively takes place at the Department of General, Visceral, and Transplantation Surgery at University Hospital of Heidelberg in Germany. Possible participants will be screened for eligibility. To be eligible for the study, participants must be 18 years or older with a resectable primary malignancy in the distal esophagus including adenocarcinoma of the esophagogastric junction type 1 and 2 with curative intention and no apparent metastases. Participants with tumor localizations above the azygos vein, emergency situations such as major bleeding or perforation as well as metastasis or infiltration into adjacent tissue are excluded.

All eligible participants will be informed about either operation technique, as well as their potential benefits and side effects. Written informed consent will be obtained. Only patients who sign the informed consent form will be included. Reasons for exclusion from the MIVATE trial will be documented and explained in the screening form. Thereafter, patients will be randomized to the intervention arm (MIE with linear stapled anastomosis) or the control arm (OE with circular stapled anastomosis). For the duration of the treatment until discharge, no concomitant interventions besides the in-house standards and study-related interventions are allowed.

### Outcome measures

**Fig. 1:**
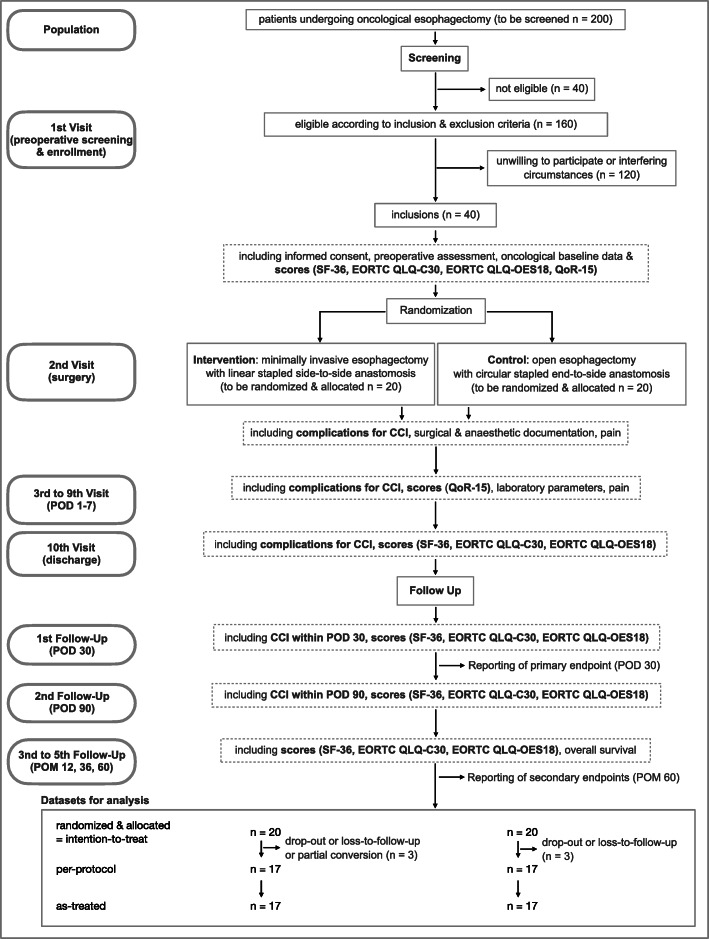
Study design flow chart. CAT EORTC QLQ-C30, Computerized adaptive test European Organization for Research and Treatment of Cancer Quality of Life Questionnaire Core 30; CAT EORTC QLQ-OES18, Computerized adaptive test European Organization for Research and Treatment of Cancer Quality of Life Questionnaire for Esophageal Cancer; CCI, Comprehensive Complication Index coding complications and related interventions according to the Dindo-Clavien classification; ICU, intermediate care unit; POD, postoperative day; POM, postoperative month; QoR-15, Quality of Recovery 15; SF-36, Short-Form 36. Only the most defining and relevant aspects of visit and follow-up documentation are indicated. For a more extensive list, please refer to Table 1 (trial visits)

During the MIVATE trial (Fig. 1), participants will be monitored before surgery, intraoperatively, on postoperative days (POD) 1–7 and on the day of discharge. Follow-up will be conducted on POD 30, as well as 3 months, 12 months, 36 months, and 60 months postoperatively. During follow-up, patients will always complete a professionally administered questionnaire. Demographic and baseline clinical data, intraoperative findings, and postoperative results will be recorded. To enhance participant retention and to avoid loss to follow-up, patients will be called during the follow-up period to remind them of scheduled visits and to arrange appointments. When a patient is not able to participate in a follow-up visit in person, questionnaires will be administered by telephone interview. Informed consent will be obtained and trial data will be collected by trained assessors by using CRFs and established questionnaires,

#### Primary endpoint

The primary endpoint will be postoperative morbidity assessed as the comprehensive complication index (CCI) within 30 days after index operation [22], which enables to compare the severity of postoperative complications [23].

Postoperative morbidity is defined as any deviation from the normal postoperative course according to the Dindo-Clavien classification [24]. Specifically, it includes anastomotic insufficiency or loss of anastomotic integrity verified by either CT scan with detection of contrast agent externally from the anastomosis within the abdominal or thoracic cavity, endoscopy, or the detection of methylene blue in the drainage after oral application. Also, it includes pneumonia with radiological verification of pneumonic infiltrates and a minimum of 3 of 4 possible symptoms including a body core temperature above 37.5 °C, purulent expectoration, leucocyte count above 12,000 or below 4500/ml, or increased C-reactive protein (CRP) levels. Postoperative complications also include pancreatic fistula, which is defined by the International Study Group for Pancreatic Surgery (ISGPS) as the “drain output of any measurable volume of fluid with an amylase level > 3 times the upper limit of institutional normal serum amylase activity, associated with a clinically relevant development/condition related directly to the postoperative pancreatic fistula.” The former grade A fistula is now called a “biochemical leak,” as it has no clinical relevance. Fistula grades B and C are defined more precisely. Grade B requires drains that are either left in place > 3 weeks or repositioned. Grade C requires reoperation or leads to single or multiple organ failure [25, 26]. Recommendations for reporting of complications after esophagectomy will be obeyed in order to improve comparability with future trials [27]. Other aspects are postoperative bleeding with a hemoglobin relevant decrease beyond 3 g/dl or the necessity for transfusion of erythrocyte concentrates due to bleeding into the abdominal or thoracic cavity. Wound healing disorders with special wound treatment, abscess, and lymphatic fistula caused by damage to the lymphatic system with leakage of chyle fluid into the cavities (defined as a milky-colored fluid from a drain, drain site, or wound on or after POD 3, with a triglyceride content ≥ 110 mg/dL respectively ≥ 1.2 mmol/L) also accounts for postoperative morbidity [28, 29]. Further aspects are tracheal injuries with fistula between the esophagus and trachea and loss of tracheal integrity as well as radiologically confirmed deep leg vein thrombosis and pulmonary embolism. Acute kidney failure in direct context to surgery defined as a doubling of plasma creatinine levels or necessity for hemodialysis as well as stroke and myocardial infarction are further criteria included in postoperative morbidity.

The CCI will be reported with mean and standard deviation.

#### Secondary endpoints

Secondary endpoints can be separated into short-term endpoints and long-term endpoints. Short-term endpoints include operation time, length of hospital stay, duration of stay on intensive or intermediate care unit (ICU), postoperative recovery assessed with QoL-15, postoperative pain assessed with the visual analog scale (VAS), the necessity for vasopressor agents for circulatory support, length of single-lung ventilation, number of days with invasive ventilation, fluid management, postoperative demand for analgesic drugs, and levels of acute-phase proteins in the serum. The successful adherence to an already established fast-track protocol with several sub-categories will also be a secondary endpoint (Additional Tables 1 and 2) [3, 30]. Short-term oncological endpoints are number of removed lymph nodes and rate of R0-resections.

Long-term endpoints are QoL and oncological outcomes such as disease-free-survival, rate of local recurrence and overall-survival. QoL will be assessed with different questionnaires. SF-36 and CAT EORTC QLQ-C30 measure general aspects of health with scores ranging from 0 to 100 and with higher scores representing better well-being. CAT EORTC QLQ-OES18 assesses several aspects of esophageal function, ranging from 0 to 100 with lower scores indicating better function [31].

Extended details of the secondary endpoints can be found in Table 1. Several other scoring systems from other institutions have been included in the design of the MIVATE trail such as the surgical site infection classification according to the CDC (Center for Disease Control and Prevention) (https://www.cdc.gov/hai/ssi/ssi.html) [32], the ASEPSIS score for wound infection [33], and the standardization of data collection for complications associated with esophagectomy from the Esophagectomy Complications Consensus Group (ECCG) [27].

**Table 1:** Trial visits

Activity and documentation	Visit 1 (screening)	Visit 2 (surgery)	Visits 3–9 (POD 1–7)	Visit 10 (discharge)	1. Follow-up (POD 30)	2. Follow-up (POD 90)	3.-5. Follow-up (POM 12, 36, 60)
Inclusion and exclusion criteria and informed consent	x						
Biometric data (*1)	X						
Medical history and preoperative assessment (*2)	x						
Oncological baseline data (*3)	x						
Pain with VAS and analgetic management	x	x	x				
ASA, ECOG, Revised Cardiac Risk Index, lung function	x						
Body weight	x				x	x	x
Randomization	x						
Surgery		x					
Surgical analgetic anesthetic documentation (*4)		x					
**Documentation of complications:**							
**Dindo-Clavien for CCI within 30 POD**			x	x	x		
Dindo-Clavien for CCI within 90 POD			x	x	x	x	
further complications (*5)			x	x	x	x	x
**scores**							
QoR-15	x		x (on POD 7)	x	x	x	x
SF-36	x			x	x	x	x
EORTC QLQ-C30 and EORTC QLQ-OES18	x			x	x	x	x
**Perioperative course**							
Length of (single-lung-) ventilation and analgetic extubation		x					
Epidural catheter management		x	x				
Hemodynamic medication		x					
Fluid management		x					
Blood gas analysis		x					
Blood count and CRP	x		x (selectively)				
Catheters (peridural, urine, central venous)		x					
**Postoperative course**							
Adherence to fast track protocol		x	x				
Gastric tube and drainage management		x	x				
Mobilization			x				
Vegetative functions (passing stool)			x				
Wound healing/infection			x				
Diet		x	x	x			
Length of hospital stay and discharge				x			
**Oncological and specific long-term**							
Histopathological data (*6)				x			
GI symptoms (*7)					x	x	x
Adjuvant therapy					x	x	x
Disease-free survival					x	x	x
Local recurrence					x	x	x
Progression-free survival					x	x	x
Overall survival					x	x	x

(*1) Including age, body height, and sex

(*2) Including medication and previous surgical intervention

(*3) Including first diagnosis, cTNM status, location relative to Z-line, and neoadjuvant chemo- or radiotherapy

(*4) Including surgeons, experience, procedure, operation time, anastomosis, complications, conversion, and drainages

(*5) Including anastomotic leak, conduit necrosis, chyle leak, and vocal cord injury according to ECCG and surgical site infection according to CDC

(*6) Including entity, pTNM, grading and resection status, number of lymph nodes retrieved, and number of tumor positive lymph nodes

(*7) Including dysphagia and reflux

*ASA* American Society of Anesthesiologists classification, *CCI* Comprehensive Complication Index coding complications and related interventions according to the Dindo-Clavien classification, *CDC* Center for Disease Control and Prevention, *CRP* C-reactive protein, *ECCG* Esophagectomy Complications Consensus Group, *ECOG* Eastern Cooperative Oncology Group score, *EORTC QLQ-C30* European Organization for Research and Treatment of Cancer Quality of Life Questionnaire Core 30, *EORTC QLQ-OES18* Computerized adaptive test European Organization for Research and Treatment of Cancer Quality of Life Questionnaire for Esophageal Cancer, *POD* postoperative day, *POM* postoperative month, *QoR-15* Quality of Recovery 15, *SF-36* Short-Form 36, *VAS* visual analog scale of pain. The primary endpoint is displayed in gray

### Standardized therapy and trial interventions

OE, hybrid esophagectomy, and totally MIE with circular stapled anastomosis are established in single high-volume centers and described to have similar oncological outcomes [14, 34, 35]. In the present study, the singularizing aspect is the linear side-to-side stapled anastomosis derived from bariatric surgery where it is established as a technique with excellent risk-benefit ratio and low anastomotic stricture rate compared to circular stapled and hand-sewn anastomosis [34, 35].

In the present study, for both interventions intubation is done with a double lumen tube and patients receive antibiotic prophylaxis perioperatively with Ampicillin-Sulbactam (3 g single-shot) or other in case of allergies. Surgery starts with the abdominal part.

In case of open surgery, the patient is placed in “Crawford” position. After median laparotomy, the surgeon performs inspection of the abdominal cavity to ensure the absence of metastases and peritoneal carcinomatosis. Subsequently, the bursa omentalis is opened through incision of the gastrocolic ligament. After dissection of the Aa. gastricae breves with preservation of gastroepiploic arcade, preparation is performed up to the splenic hilus and to the left crus. The gastric conduit is formed through the application of linear staplers (Endo-GIA stapler with Tri-Staple Technology® from Medtronic®, Dublin, Ireland) alongside the lesser curvature and the separation of the distal esophagus. Additional sutures secure the integrity of the staple line. Lymphadenectomy (LAD) is following alongside the splenic artery (station 11) and around the coeliac trunk and the common hepatic artery (stations 8–9). Cholecystectomy is usually performed. LAD is continued in transhiatal direction towards both crura of the diaphragm into the lower mediastinum. During this process, both pleurae are opened and partly resected. Ventral border for LAD is the pericardium, while dorsal resection is limited by the aorta. The gastric conduit is placed transhiatally for later transposition and the abdomen is preliminary closed.

For the thoracic part, the right lung is vented and a right-lateral thoracotomy is performed. The azygos vein is separated and LAD is performed following the very same. The thoracic duct is clipped and separated and LAD is performed down the aorta. Infratracheal lymph nodes are resected and the esophagus is proximally transected with a linear stapler. The specimen is retrieved and sent for pathohistological assessment. Frozen sections are usually obtained from the proximal resection line.

The formation of an end-to-side esophagogastrostomy starts with the transection of the esophagus at the resection line and the insertion of the circular stapler anvil with the size chosen depending on the anatomic situation. The size of the anvil is usually 25 or 28 mm (preferably) in diameter and as large as possible in order to prevent strictures. The anvil is positioned and fixed with a purse-string suture. The gastric conduit is now moved towards the anastomotic site in the thoracic cavity and incised distally in order to insert the circular stapler shaft. After the stapling process for the creation of the end-to-side anastomosis, the inserting incision on the ventral side of gastric conduit is closed with another linear stapler. A toluidine blue test is performed to check for anastomotic integrity. Finally, a Robinson drainage (16 Chr) is used as a target drainage and placed in front of the anastomosis followed by bilateral Bülau drains (24 Ch). The thoracotomy is closed with sutures and an EasyFlow-Drainage can be placed intraabdominally close to the upper pancreatic margin upon preference of the surgeon. The laparotomy is then finally closed with sutures.

The technique of MIE used within this study has recently been shown in detail elsewhere [36]. The patient is first placed in “French” position. After left paramedian skin incision and the insertion of a 12-mm optical trocar, a pneumoperitoneum is established with a pressure of 15 mmHg. After insertion of further trocars (2 × 5 mm, 2 × 12 mm) as well as a subxiphoidal Nathanson liver retractor, surgery continues as described above. Single button sutures are used on the crossing sites of the linear stapler lines as opposed to the continuous sutures covering the whole linear stapler lines in the open approach and facilitate manipulation of the gastric conduit. Placement of an abdominal drain is optional. For the thoracic part, the patient is placed in left lateral decubitus position. After insertion of a 12-mm optical trocar below the right scapula, a pneumothorax is established with a pressure of 8 mmHg and further working trocars (3 × 12 mm) are placed under permanent visual control. Further surgical steps are identical to the open approach. The specimen is then put into an 800-ml retrieval bag and evacuated via a 4-cm incision along the 11th intercostal space.

The formation of a side-to-side esophagogastrostomy starts with the incision of the esophageal stump in the middle of the linear stapler line under continuous counterpressure through a 42-French-esophageal-tube. The gastric conduit is positioned atraumatically by exclusively moving it via the vicryl sutures. It is now incised 5 cm away from the oral stapling margin and a 45-mm linear tristapler is inserted into both incisions entering only 3 cm into both luminae at the same time. The incomplete insertion of the stapler leaves a length of 2 cm between distal staple line and anastomosis. After the stapling process, the remaining aperture is closed with a two-layer continuous suture with Stratafix® (Ethicon Endo-Surgery®, Cincinnati, Ohio, USA). Thoracic drains are inserted as in the open approach.

For standardization purposes, surgeons performing this type of surgery for following trials must have surpassed the learning curve which is described to last until at least 50 esophagectomies with the specific technique [37, 38]. This is clearly relevant in order to reduce surgeon-related influences as it could be shown by Nimptsch in 2018 that the mortality rate after esophageal surgery was lower in centers with high case numbers compared to those with very low case numbers with an OR[CI] of 0.50 ([0.42; 0.60]). Among patients who had complications, the in-hospital mortality[CI] was 12.3% [11.1; 13.7] in hospitals with very high case numbers compared to 20.0% [18.5; 21.6] in hospitals with very low case numbers, indicating that the quality of treatment for patients undergoing esophageal resection could be improved if more patients were treated in hospitals with high case numbers [2]. Therefore, an expertise-based design was chosen for the MIVATE trial and only the single most experienced surgeon of the center for the respective technique is performing trial surgeries.

### Modification of the protocol

The current protocol version from March 2020 is the protocol the trial was initiated with (protocol version 1.0). In case of protocol amendments, these will be submitted to the ethics committee for approval and no further recruitments will take place until the modifications are accepted.

### Assessment of safety and termination criteria

All adverse events will be documented and analyzed because complications form the study’s primary endpoint in the form of the CCI. Participants will be excluded from the study if they withdraw their consent to participate in the trial. A participant may withdraw consent at any time without explanation and without affecting further medical care. The principal investigator may terminate the trial at any time in consultation with the key research associates and the biostatistician. Possible reasons for termination include high morbidity or mortality rates and any indication of potential health hazards caused by either the study treatment or external factors. In case of intraoperative complications in the minimally invasive group, there might be the need to convert to open surgery. In the case of intraoperatively identifying advanced and irresectable disease, there will be a change to palliative treatment strategies. There are no other criteria for modifying or discontinuing treatment.

### Randomization and blinding

A random allocation sequence has been generated by computer through block-randomization prior to the start of the trial by an independent third party. These allocations have then been put in sequentially numbered, sealed, opaque envelopes and are opened prior to the patient’s surgery. Block sizes of 4, 6, and 8 were used in a variable order. After informed consent, patients will be enrolled by trial-trained physicians and randomized to the intervention or control group on the day before surgery. Allocation is performed by opening these envelopes containing cards displaying “Endoscopic” or “Open.

Blinding of study contributors [39]: No attempt will be done to blind patients and the access sites will be covered with standard wound dressings until discharge. The blinding of the operating surgeon is not possible. The severity of pain and rescue analgesic will be evaluated by anesthesiologists otherwise not involved in the study (data collectors). All cases are reviewed regarding the primary endpoint by a neutral outcome assessor. The statistical analysis will be performed according to the outlined protocol; no additional attempts are made to blind the statistician as this will have no influence on the predefined statistical analysis of previously recorded and saved data.

### Data management

All data will be collected and recorded in case report forms (CRFs) by an investigator before transfer to the data management center. Personal information about potential and enrolled participants will be collected, shared, and maintained with third party only after pseudonymization in order to protect confidentiality. All demographic and baseline clinical data, as well as primary and secondary outcome measures, will be recorded in the CRF. To promote data quality, there will be automated checks for double data entry and value ranges. To ensure patient confidentiality, the CRF for each patient will be given an anonymous allocation number. We will obtain permission to continue follow-up and data collection in the event of withdrawal from the study. The responsible investigator must review and sign all completed CRFs.

### Statistical methods

#### Sample size

The sample size calculation is based on the primary endpoint “postoperative morbidity measured with the CCI until POD 30”. MIVATE is a superiority trial. The null hypothesis claims that the minimally invasive approach is not superior to open surgery in terms of CCI. The alternative hypothesis claims that the minimally invasive approach is superior to open surgery in terms of CCI within POD 30. A decrease of the CCI by 10 points between the minimally invasive and open group is considered relevant by patients and clinicians and a standard deviation of 10 is assumed based on in-house data leading to an effect size *d* of 1.0. There are no studies explicitly indicating the standard deviation of the CCI after esophagectomy.

Based on a *t* test with a two-sided significance level of *α* = 0.05, a sample size of *n* = 34 patients (17 per group) is required in the analysis dataset in order to achieve a power of 80% (calculations performed with Prism 8.0, G*Power and SPSS). To compensate for early trial termination, drop-outs, and loss-to-follow-ups, further 15% of patients will be randomized leading to a total randomization and allocation size of 40 patients (20 per group). The number of patients to be screened and assessed for eligibility (*n* = 200; 100 per group) was calculated with an assumed 20% of exclusions due to exclusion criteria and a 75% of interfering circumstances (200 × 0.8 × 0.25 = 40; 20 per group). These interfering circumstances are due to the fact that MIVATE is an expertise-based trial which only allows the institution’s single best surgeon for each allocated technique. Consequently, both surgeons have to be available at the time of randomization which is not possible in 3 of 4 cases.

Screened and assessed for eligibility (*n* = 200; 100 per group)

Allocated to trial and analyzed for intention-to-treat dataset (*n* = 40; 20 per group)

Per-protocol and as-treated dataset (*n* = 34; 17 per group)

There will be regular meetings by the investigators every 3 months in order to optimize recruitment and ensure sufficient enrolment.

#### Statistical analysis

Superiority of the intervention versus the control will be assessed using a two-sided *t* test. The primary analysis will be based on the intention-to-treat population. If values do not display normal distribution, the Mann-Whitney *U* test will be used. A per protocol and an as treated set will be evaluated as a sensitivity analysis. Missing data for the primary outcome variable will be replaced by using imputation [40]. The primary analysis will test the following hypotheses:

- H_0_: CCI_open_ ≤ CCI_minimally invasive_
- H_1_: CCI_open_ > CCI_minimally invasive_

CCI_open_ and CCI_minimally invasive_ are the mean of the indices of both groups at different time points.

All secondary outcomes will be evaluated descriptively, and descriptive *p* values are reported. All analyses will be done using SPSS, SAS, and R. A detailed statistical analysis plan is developed prior to the analysis of the trial results in order to guarantee for neutrality during the analysis.

There will be no interim analysis for the primary endpoint. However, the primary endpoint will be analyzed as soon as all relevant data has been obtained. There will be interim analyses for the secondary endpoints.

## Discussion

Localized esophageal carcinoma can be treated curatively with surgery, but esophagectomy remains challenging with considerable morbidity and mortality, an extensive recovery period as well as impairment of QoL. In comparison to other major gastrointestinal surgeries, open esophagectomy is associated with high rates of complications of up to 34% of pneumonia as described by Biere et al. in 2012 [41] and up to 19.4% of anastomotic leakage, as well as 15.8% of recurrent laryngeal nerve palsy as described by Booka et al. in 2015 and Asaka et al. in 2019 in case of cervical anastomosis [42, 43]. The overall pulmonary complications are described to be as high as 40.5% for OE compared to 9.5% for MIE (*p* = 0.004) in some studies [44]. Especially pneumonia has a significant negative impact on survival (*p* = 0.035) with a multivariate HR of 1.456 (([CI] [1.020; 2.079]) *p* = 0.039). Therefore, strategies to prevent pneumonia after esophagectomy are assumed to improve postoperative outcomes [42]. The MIVATE trial is the first RCT to compare total MIE versus OE in an expertise-based approach. It focuses on the difference of postoperative complications within 30 postoperative days which are mainly caused by anastomotic insufficiencies and pulmonary impairments. The primary endpoint is the CCI within postoperative day 30 and therefore a highly objective and standardized parameter that will also be suitable in performing sample size calculations for further randomized multicenter trials and thus also enabling large-scale meta-analyses.

The MIVATE trial consist of a complex intervention with two subinterventions: The first and main subintervention is the comparison of the total minimally invasive surgical approach (MIE) compared to the open approach (OE), whereas the second subintervention is the use of two different anastomotic techniques that are specified for each group. The linear stapled anastomotic technique is only used in the MIE group, whereas the circular stapled approach is only used in OE. This is mainly due to practical reasons since the linear stapling technique is facilitated by the minimally invasive access with trocars whereas this technique is more cumbersome in open approach due to angulation and access difficulties. On the other hand, the circular stapling technique is more practical in open surgery whereas in minimally invasive surgery this proves more difficult. This combination of two different steps of intervention leads to the problem that identified differences between the groups can possibly not be ascribed to one specific interventional aspect. However, while complex interventions are not necessarily suited to explain effects mechanistically—which is not the scope of this trial—they are recognized to be more sensitive to differences between groups and to more effectively reflect on reality. Consequently, the advantage of this complex intervention is its ability to screen for several interventional steps at once resulting in an increased probability of difference detection as well as its representative validity of esophageal surgery representing regularly performed operation methods and thereby increasing the relevance and external validity of this trial. Yet, there will be descriptive subgroup analysis to identify potential major influencing factors.

Another strength of the MIVATE trial is the extensive surgical standardization. There is no surgical heterogeneity within the groups as every single patient of one group is treated by the same highly experienced surgeon according to the aforementioned description of expertise-based principles. This reduces bias by ensuring that every patient gets the best surgical expertise available and avoids both the “problem of the poor control group” [45] and poor recruitment. Furthermore, patients follow a highly standardized fast track protocol for postoperative recovery, of which the adherence will be documented and reported.

The MIVATE trial specifically addresses the issue of short-term complications in comparing OE and MIE with two different anastomotic techniques with the intention of making a contribution to an optimized treatment strategy for esophageal cancer. Long-term oncological outcomes will have to be evaluated in further multicenter trials with adequate power.

In summary, this monocenter trial will evaluate the difference between OE and total MIE focusing on postoperative complications. The MIVATE trial will provide further evidence of optimal technique for oncologic esophagectomy comparing the open and total minimally invasive approach. The two singularizing aspects of this trial are the linear stapled anastomosis for total MIE as well as the expertise-based approach. The findings will serve as a basis for conducting multicenter RTCs to evaluate which procedure is best for patients requiring esophagectomy in order to further optimize outcomes and reduce complications.

## Trial status

The first patient was randomized in March 2019 and recruitment is planned for 3 years. Consequently, recruitment is planned to be completed in March 2022. At the time of the protocol submission (April 2020), 10 of 40 patients (25%) had been randomized. On the date of submission (11 April 2020), this protocol is in its first version.

## Supplementary Information

**Additional file 1.** SPIRIT checklist.

**Additional file 2: Additional Table 1.** Fast-Track Esophagectomy Protocol on Esophageal Cancer Patient.

**Additional file 3: Additional Table 2.** STOMA diet levels.

**Additional file 4.** Clinical Report Form MIVATE.

## Data Availability

The full protocol, participant-level dataset, and statistical code are available from the corresponding authors on reasonable request.

## Abbreviations

ADL: Activities of daily living
ASA: American Society of Anesthesiologists classification
CAT EORTC QLQ-C30: Computerized adaptive test European Organization for Research and Treatment of Cancer Quality of Life Questionnaire Core 30
CAT EORTC QLQ-OES18: Computerized adaptive test European Organization for Research and Treatment of Cancer Quality of Life Questionnaire for Esophageal Cancer
CCI: Comprehensive complication index
CDC: Center for Disease Control and Prevention
Ch: Charrière
CI: 95% confidence interval
CRF: Case report form
CRP: C-reactive protein
ECCG: Esophagectomy Complications Consensus Group
ECOG: Eastern Cooperative Oncology Group score
HR: Hazard ratio
ICU: Intensive care and intermediate care unit
IQR: Interquartile range
IV: Intravenous
LAD: Lymphadenectomy
MIE: Minimally invasive esophagectomy
NGT: Nasogastric tube
OE: Open esophagectomy
OR: Operation room
PCA: Patient controlled analgesia
POD: Postoperative day
POM: Postoperative month
PPI: Proton pump inhibitor
QoL: Quality of life
QoR-15: Quality of Recovery 15
RAMIE: Robotic-assisted minimally invasive esophagectomy
RCT: Randomized controlled trial
SAE: Serious adverse event
SF-36: Short-Form 36
SOP: Standard operating procedure
VAS: Visual analog scale
WHO: World Health organization
