# Supplementary material for: Minimally Invasive Versus open AbdominoThoracic Esophagectomy for esophageal carcinoma (MIVATE) — study protocol for a randomized controlled trial DRKS00016773
Source: Trials. 2021 Jan 11;22:41. doi: 10.1186/s13063-020-04966-z (PMC7798277; doi:10.1186/s13063-020-04966-z)
Supplement: Supplementary file 2 — Additional file 2: Additional Table 1. Fast-Track Esophagectomy Protocol on Esophageal Cancer Patient. [file 13063_2020_4966_MOESM2_ESM.docx]

**Additional Table 1 | Fast-Track Esophagectomy Protocol on Esophageal Cancer Patient.**

*Ch* Charrière**,** *ICU* intensive care and intermediate care unit, *IV* intravenous, *NGT* nasogastric tube, *OR* operation room, *PCA* patient controlled analgesia, *POD* postoperative day, *PPI* proton pump inhibitor, *SOP* standard operating procedure, *WHO* World Health organization.
