# Supplementary material for: Minimally Invasive Versus open AbdominoThoracic Esophagectomy for esophageal carcinoma (MIVATE) — study protocol for a randomized controlled trial DRKS00016773
Source: Trials. 2021 Jan 11;22:41. doi: 10.1186/s13063-020-04966-z (PMC7798277; doi:10.1186/s13063-020-04966-z)
Supplement: Supplementary file 3 — Additional file 3: Additional Table 2. STOMA diet levels. [file 13063_2020_4966_MOESM3_ESM.docx]

**Additional Table 2 | STOMA diet levels**.


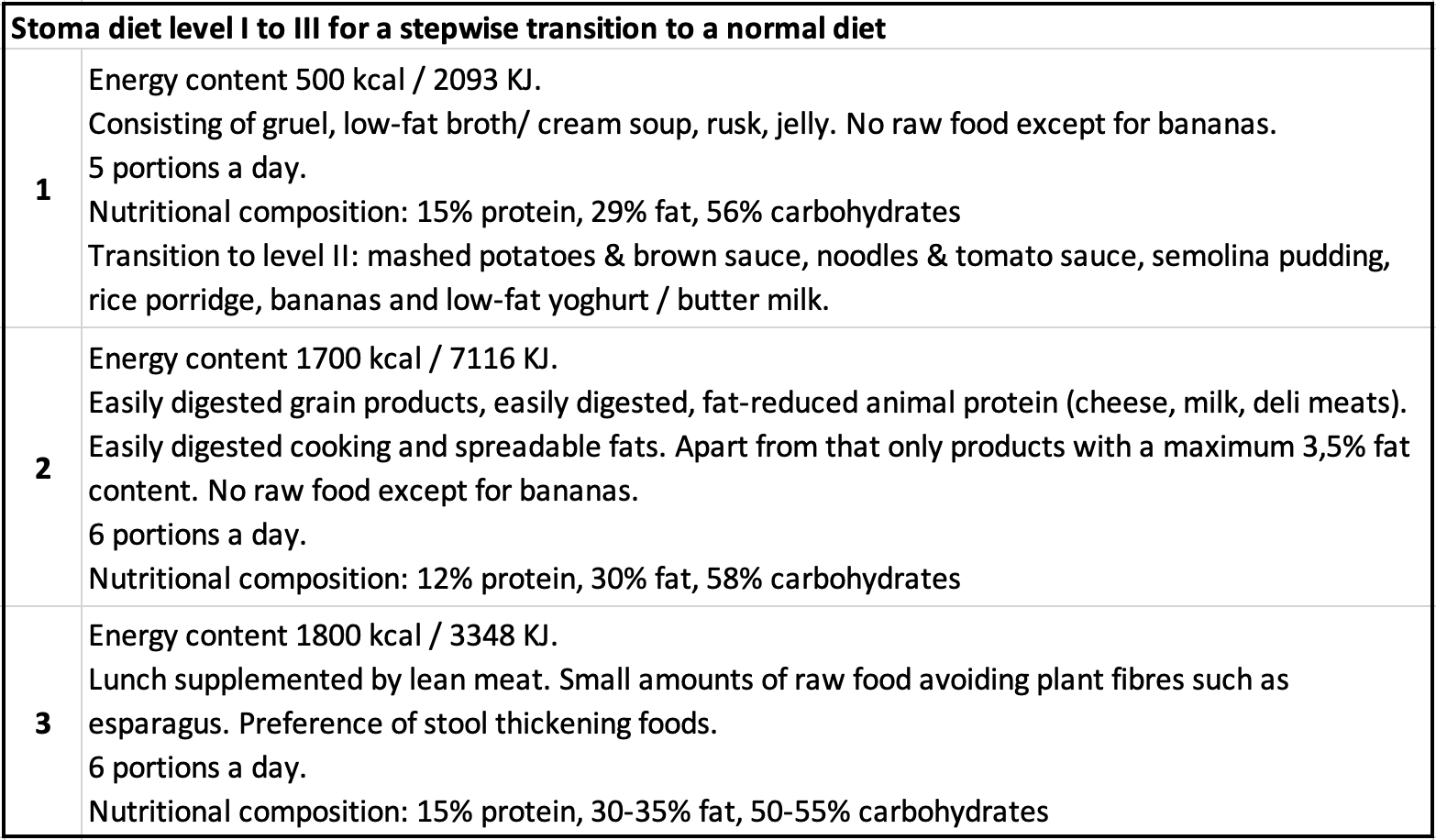


A scheme of a stepwise transition to a normal diet after visceral surgery according to in-house-standards of the University Hospital of Heidelberg.
