# Supplementary material for: Minimally Invasive Versus open AbdominoThoracic Esophagectomy for esophageal carcinoma (MIVATE) — study protocol for a randomized controlled trial DRKS00016773
Source: Trials. 2021 Jan 11;22:41. doi: 10.1186/s13063-020-04966-z (PMC7798277; doi:10.1186/s13063-020-04966-z)
Supplement: Supplementary file 4 — Additional file 4. Clinical Report Form MIVATE. [file 13063_2020_4966_MOESM4_ESM.docx]

**(MIVATE - Studie)**

Single-Centre Randomized Trial comparing Minimally Invasive Versus open Abdomino Thoracic Esophagectomy

Patientendokumentation

Patientennummer: ____/____/____

Randomisierungsnummer ____/____/____

Studienleitung: Prof. Dr. med. Beat Müller, Universitätsklinikum Heidelberg
Studienkoordinator: PD Dr. med. Felix Nickel, Universitätsklinikum Heidelberg

**Randomisierung vor der Operation:**

**0049 6221 56 36204**

# Visite 1: Screening-Visite Datum: ____/____/________

Einschlusskriterien:

Ja Nein

⬜ ⬜ Malignom des distalen Ösophagus mit Absetzungshöhe unterhalb V. azygos (inkl. AEG)

⬜ ⬜ Geplante Durchführung einer abdomino-thorakalen Ösophagektomie mit Anlage einer intrathorakalen Anastomose in kurativer Intention

⬜ ⬜ Patientenalter über 18 Jahre

⬜ ⬜ Informed Consent (Fähigkeit und Bereitschaft zur Einwilligungserklärung)

Ausschlusskriterien:

Ja Nein

⬜ ⬜ Geplante Absetzungshöhe oberhalb der V. azygos

⬜ ⬜ Notfalleingriff z.B. auf Grund von Blutung oder Perforation

⬜ ⬜ Hinweis auf Tumorinfiltration von Nachbarorganen

Um den Patienten in die Studie einschließen zu können, muss bei **allen** **Einschlusskriterien** „JA“ und bei **allen** **Ausschlusskriterien** **„NEIN**“ angekreuzt sein!

# Biometrische Daten:

Alter [Jahren]: ________ Geschlecht: ⬜ männlich ⬜ weiblich

Körpergröße [cm]: ________ Gewicht [kg]: ________

Fragebögen**:**

Lebensqualitätserfassung: SF-36 ⬜ ____________

EORTC QLQ-C30 ⬜ ____________

EORTC QLQ- OES18 ⬜ ____________

QoR-15; Quality of Recovery 15: ⬜ ____________

Schmerz-Therapie präoperativ ⬜ Ja ⬜ Nein

Wirkstoff(e) + Dosis [mg/d] ___________________________________________

___________________________________________

___________________________________________

___________________________________________

___________________________________________

Behandlungszeitraum mehr als 2 Wochen präoperativ ⬜ Ja ⬜ Nein

Falls „ja“: welche(s) Präparat(e): ____________________________________

____________________________________

____________________________________

____________________________________

____________________________________

Onkologische Daten: Erstdiagnose ____/____/________

Stadium (cTNM): ______________________________

Höhe Z-Linie (ab Zahnreihe in cm): ­­­­­­­­­­________________________

Lokalisation proximaler Tumorrand in Bezug zur Z-Linie - in cm: ­­­­­­­­­___________________

Lokalisation distaler Tumorrand in Bezug zur Z-Linie - in cm: ___________________

Neoadjuvante **Chemotherapie** ⬜ Ja ⬜ Nein

Therapieschema: __________________________________,
Anzahl durchgeführter Zyklen Chemotherapie: ____

Neoadjuvante **Radiotherapie** ⬜ Ja 🡪 Gesamtdosis [Gy]: ____________ ⬜ Nein

Vor – Operationen:

Z. n. vorheriger Oberbauch-OP ⬜ Ja ⬜ Nein

Welche: ______________________________________________________________________

Weitere abdominelle oder thorakale Vor-OPs ⬜ Ja ⬜ Nein

Welche: ______________________________________________________________________

Narkoserelevante Parameter:

Risikoklassifikation:

ASA Klasse (1 – 5): _______

WHO/ ECOG Performance Status (0 – 5): ___________

| WHO/ ECOG | Punkte |
| --- | --- |
| Normale uneingeschränkte Aktivität, wie vor der Erkrankung | 0 |
| Einschränkung bei körperlicher Anstrengung, gehfähig, leichte körperliche Arbeit möglich | 1 |
| Gehfähig, Selbstversorgung möglich, aber nicht arbeitsfähig, kann mehr als 50% der Wachzeit aufstehen | 2 |
| Nur begrenzte Selbstversorgung möglich, 50% oder mehr der Wachzeit an Bett oder Stuhl gebunden | 3 |
| Völlig pflegebedürftig, keinerlei Selbstversorgung möglich, völlig an Bett oder Stuhl gebunden | 4 |
| Tot | 5 |

Revised Cardiac Risk Index (s.u.): Gesamtpunktzahl: _____/ 6 Punkte

| **Risikofaktor** | **Ja (+1)** | **Nein (0)** |
| --- | --- | --- |
| Bekannte Herzinsuffizienz |  |  |
| Bestehende KHK |  |  |
| Zerebrovaskuläre Erkrankungen |  |  |
| Insulinpflichtiger D.m. |  |  |
| Kompensierte Nierininsuffizienz mit  Serumkreatinin > 2 mg/dl |  |  |
| Art des Eingriffs (thorakal, intraperitoneal,  suprainguinal, vaskulär) |  |  |

Lungenfunktion: Präoperative Einsekundenkapazität: FEV1 [Liter]: _________

# Randomisierung: ⬜ Ja ⬜ Nein

Wenn Nein, Grund: ____________________________________

Randomisierungszeitpunkt**: ____/____/________ Tag/Monat/Jahr**

Randomisierungsnummer**: __________________**

**Randomisierung zu: ⬜ Offenem Verfahren**

**. ⬜ Minimalinvasivem Verfahren**

SF – 36 Fragebogen (Vorgefertigter Ausdruck)

EORTC QLQ-C30


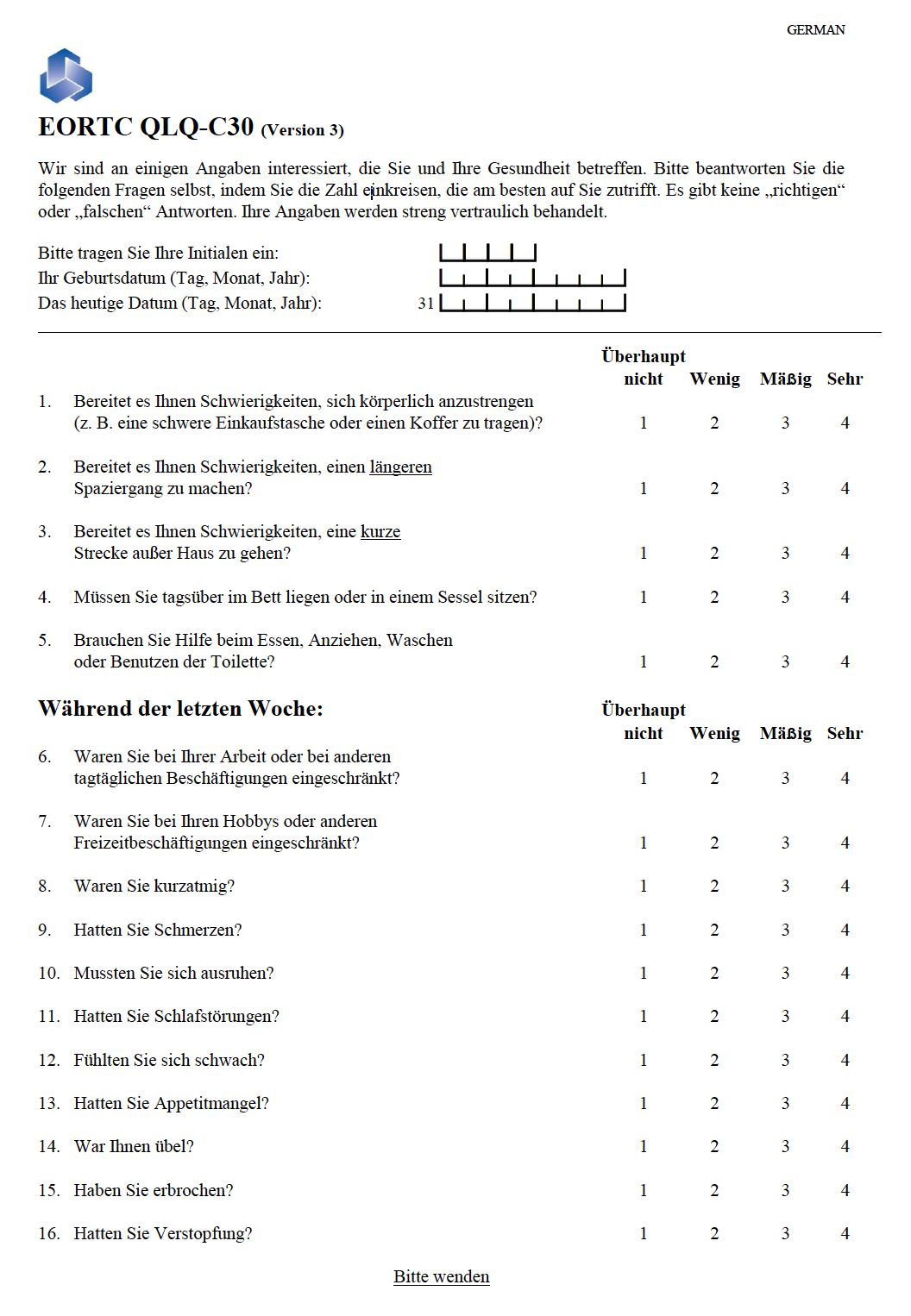


**
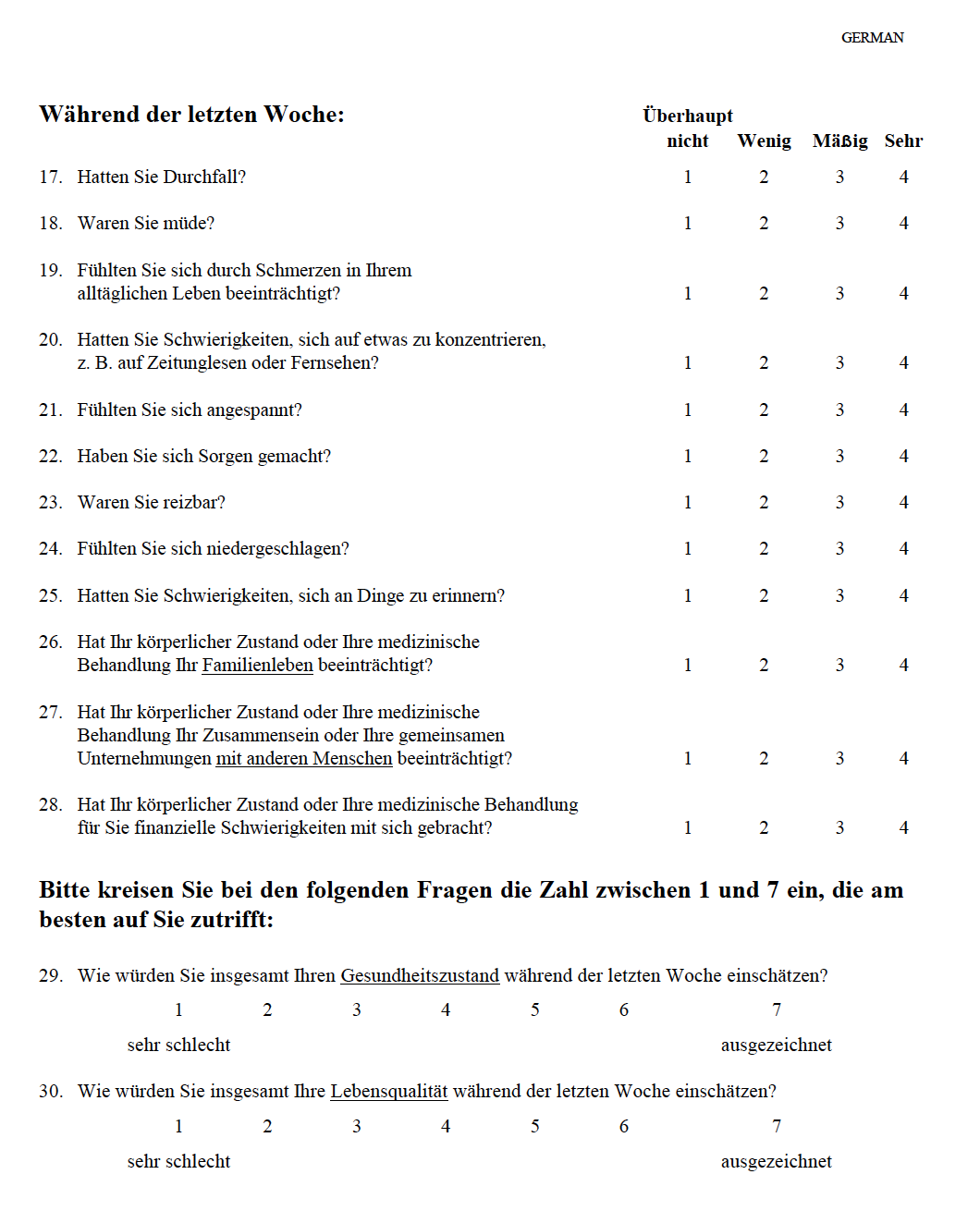
**

EORTC QLQ-OES18

**
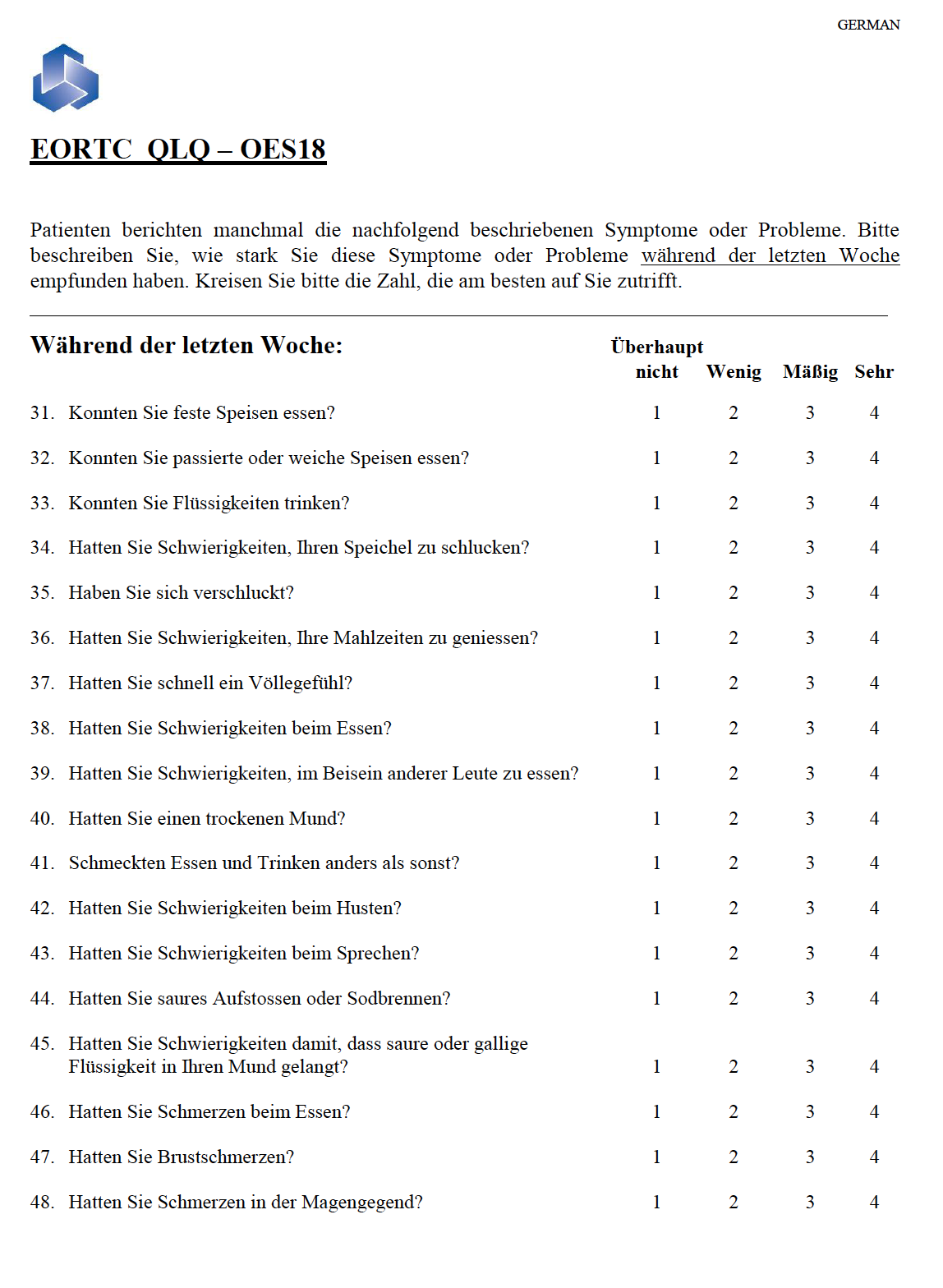
**

QoR-15; Quality of Recovery 15

**
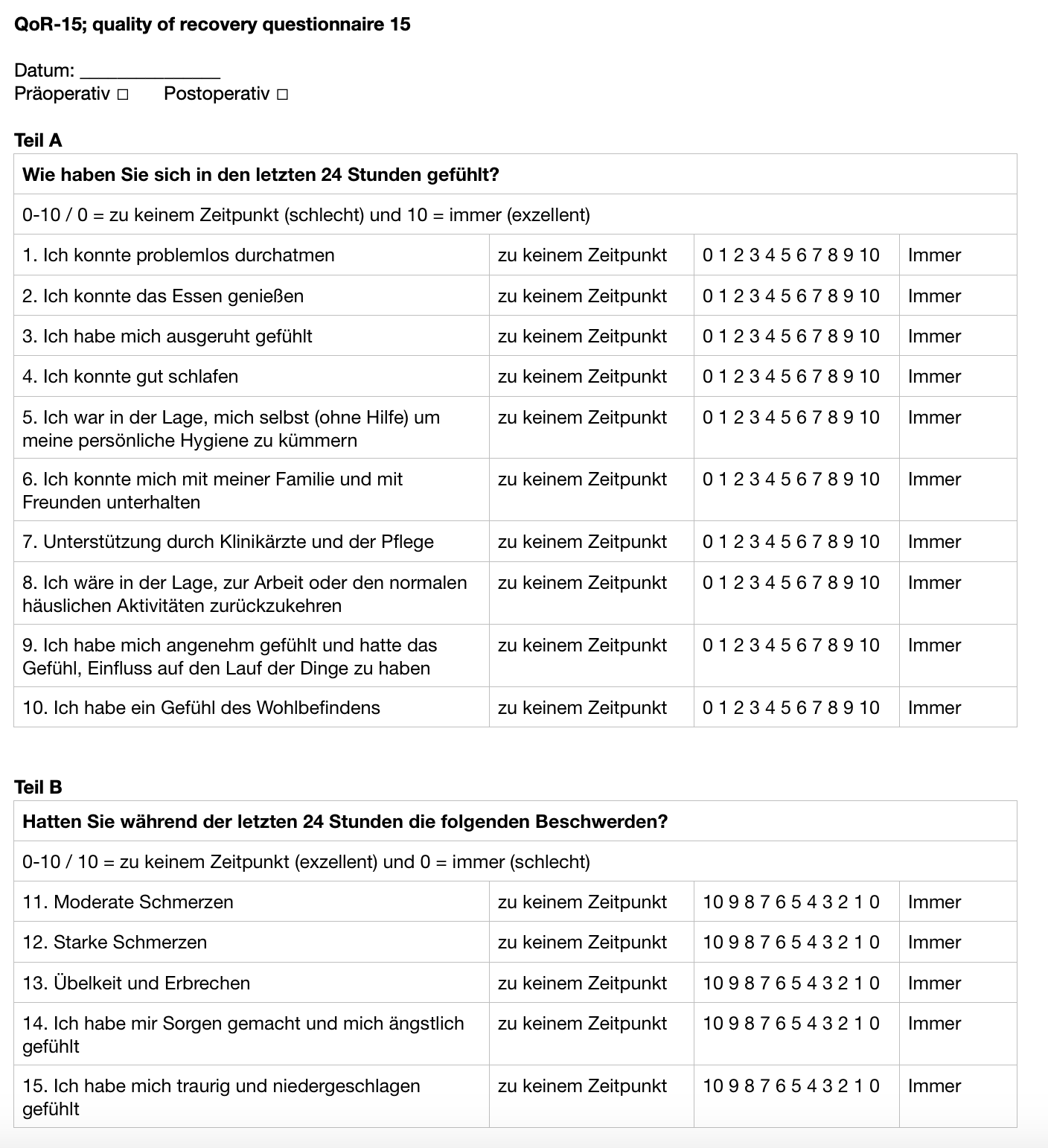
**

**Visite 2: OP – Tag Datum: ____/____/________**

Operationsteam:

Name des 1. Operateurs: ____________________________________________

Name des 2. Operateurs: ____________________________________________

| 1. Operateur  offen / minimal invasiv | Anzahl **selbst durchgeführter** Ösophagektomien zum Zeitpunkt der aktuellen OP offen / minimal invasiv | 2. Operateur  offen / minimal invasiv |
| --- | --- | --- |
| ⬜ / ⬜ | < 26 | ⬜ / ⬜ |
| ⬜ / ⬜ | 26 – 50 | ⬜ / ⬜ |
| ⬜ / ⬜ | 51 – 75 | ⬜ / ⬜ |
| ⬜ / ⬜ | 76 – 100 | ⬜ / ⬜ |
| ⬜ / ⬜ | > 100 | ⬜ / ⬜ |

| 1. Operateur  offen / minimal invasiv | Anzahl **assistierter** Ösophagektomien zum Zeitpunkt der aktuellen OP offen / minimal invasiv | 2. Operateur  offen / minimal invasiv |
| --- | --- | --- |
| ⬜ / ⬜ | < 26 | ⬜ / ⬜ |
| ⬜ / ⬜ | 26 – 50 | ⬜ / ⬜ |
| ⬜ / ⬜ | 51 – 75 | ⬜ / ⬜ |
| ⬜ / ⬜ | 76 – 100 | ⬜ / ⬜ |
| ⬜ / ⬜ | > 100 | ⬜ / ⬜ |

Operationsverfahren:

| **Bauchschnitt (OP-Beginn)** | Uhrzeit: _______________ |  |
| --- | --- | --- |
| **Umlagerung** | Uhrzeit Beginn: __________ | Uhrzeit Ende: _____________ |
| **Thorakaler Schnitt (Beginn thorakaler Teil)** | Uhrzeit: _____________ |  |
| **Bauchdecken Verschluss (OP-Ende)** | Uhrzeit: ___________ |  |

**Abdomineller Teil:**

⬜ offen ⬜ thorakoskopisch ⬜ konvertiert (Grund: ____________________________)

Besonderheiten: ____________________________________________

**Thorakaler Teil:**

⬜ offen ⬜ thorakoskopisch ⬜ konvertiert (Grund: ____________________________)

Besonderheiten: ____________________________________________

**Anastomose**:

⬜ Zirkulär Stapler ⬜ Linear Stapler; Besonderheiten: _________________________________

**Anästhesie-Protokoll:** (Bitte vom Anästhesieformblatt übertragen)

Epiduralkatheter:

Anlage eines thorakalen Epiduralkatheters vor der Narkoseeinleitung ⬜ ja ⬜ nein

Grund, falls „nein“: __________________________________

Intraoperative Parameter:

| Startzeitpunkt | [Uhr] |  |
| --- | --- | --- |
| Endzeitpunkt | [Uhr] |  |
| Pausen: Ja/ Nein |  |  |
| Start Pause(n) | Pause 1: | Pause 2: |
| Ende Pause(n) | Pause 1: | Pause 2: |

Ein-Lungenventilationszeit:

Herzrhythmusstörung ⬜ ja ⬜ nein Bezeichnung: _____________________________

Hämodynamisch relevante Medikamente:

Noradrenalinbedarf (Arterenol):

| **Konzentration** | **Perfusor (**Gesamtgabe in **[ml])** | **Bolus (**Gesamtgabe in **[ml])** |
| --- | --- | --- |
| 1 : 100 |  |  |
| 1 : 10 |  |  |
| 0,02 |  |  |
| 0,2 |  |  |
| **Summe in [ml]** |  |  |

Akrinorbedarf:

| **Konzentration** | **Gesamtgabe** in [**ml**] |
| --- | --- |
| 2 : 10 |  |
| Andere: ________ |  |

Weitere hämodynamisch relevanten Medikamente: ⬜ ja ⬜ nein

. - Dobutrex: Gesamtmenge in [ml]: ____________

- Andere: ____________________, Gesamtmenge in [ml]: ___________

Fluid Management (intraoperativ):

Blutverlust: Intraoperativer Blutverlust in [ml]: _________________

Volumenersatz:

Kristalloide [Menge in ml]: _____________________

Kolloide: Welche: _____________________, [Menge in ml]: ___________

. Welche: _____________________, [Menge in ml]: ___________

Blutersatzprodukte ⬜ nein ⬜ Ja 🡪 ⬜ EK, Anzahl: ________________

⬜ Plasma, Anzahl: ____________

⬜ TK, Anzahl: ________________

Extubation im OP: ⬜ ja ⬜ nein;

Grund, wenn nein: ________________________________, nicht extubiert mit FIO_2_: ________.

Zeitpunkt der Extubation [Uhr]: ___________, O_2_ Bedarf extubiert: _____Liter O_2_/min.

Blutgasanalysen:

Dokumentiert werden 4 BGA’s:

1. Wert (1) nach der Einleitung im OP – Saal bei 2 Lungenventilation (2-LV)

2. Wert (2) 10 Minuten nach Beginn der Einlungenventilation (1 – LV)

3. Wert (3) zu späterem Zeitpunkt der 1 – LV

4. Wert (4) nach Öffnung des blockierten Lungenflügels (2 – LV)

- SIEHE AUSDRUCK

Perioperatives Management:

**Katheterausstattung**:

PDK ⬜ Ja ⬜ Nein 🡪 Grund: ____________________________________

Urinableitung ⬜ Ja ⬜ Nein 🡪 Grund: ____________________________________

ZVK ⬜ Ja ⬜ Nein 🡪 Grund: ____________________________________

**Ernährung**: schluckweises Trinken klarer Flüssigkeit ⬜ Ja ⬜ Nein

Grund angeben, falls „nein“___________________________________________

**Schmerztherapie**:

PDK: Vorhanden: ⬜ Ja 🡪 Dosis ⬜ Nein 🡪 Grund: _____________________________

⬜ Naropin 0,2% (andere Dosierung: _____)

⬜ Sufentanil 0,5 ug/ml (andere Dosierung: _____)

Laufrate [ml/h]: _______ Uhrzeit: _______ Uhr

Zusätzliche Schmerzmedikation nach Analgesie – SOP ⬜ Ja ⬜ Nein

Wenn ja, welche + Menge/ Tag: _____________________________________

_____________________________________________________________

_____________________________________________________________

_____________________________________________________________

**Bei Abweichungen/ Besonderheiten bitte präzisieren**: ___________________________

________________________________________________________________________

Fast-Track Protokoll (vgl. Anhang 8. SOP – Leitfaden für postop. Vorgehen nach Ösophagektomie):

Hier bitte Einhaltung und Abweichungen vom SOP während des Krankenhausaufenthaltes eintragen!

| **Aktion** | **Zeitpkt. Geplant** | **Zeitpunkt Durchgeführt** | **Anmerkung/ Grund der Verzögerung** |
| --- | --- | --- | --- |
| **Verlegungen** |  |  |  |
| Verlegung auf Allgemeinstation | POD 1 __/__/___ |  |  |
|  |  |  |  |
|  |  |  |  |
| **Ernährung** | Geplanter Zeitpunkt | Durchgeführter Zeitpunkt | Anmerkung/ Grund der Verzögerung |
| Beginn Trinken klarer Flüssigkeit | POD 1 __/__/___ |  |  |
| Beginn NutriFlex Ernährung | POD 2 __/__/___ |  |  |
| Beginn Kostaufbau ^[[1]](#footnote-1)^ (TSS/ Stoma I) | POD 5 __/__/___ |  |  |
| Entfernung der Magensonde | POD 5 __/__/___ |  |  |
| Steigerung Kostaufbau (Stoma II) | POD 6 __/__/___ |  |  |
| Ende parenterale Ernährung | POD 6 __/__/___ |  |  |
| Beginn Wunschkost | POD 7 __/__/___ |  |  |
| Ernährungsberatung | POD 7 __/__/___ |  |  |
|  |  |  |  |
|  |  |  |  |
|  |  |  |  |
|  |  |  |  |
| **Drainagen** | Geplanter Zeitpunkt | Durchgeführter Zeitpunkt | Anmerkung/ Grund der Verzögerung |
| Entfernung abdominelle Drainage | POD 1 __/__/___ |  |  |
| Entf. Linksseitige Thx – Drainage | POD 1 __/__/___ |  |  |
| Entf. Rechtsseitige Thx - Drainage | POD 2 __/__/___ |  |  |
| Entf. Zieldrainage | POD 5 __/__/___ |  |  |
|  |  |  |  |
|  |  |  |  |
|  |  |  |  |
| **Katheterausstattung** | Geplanter Zeitpunkt | Durchgeführter Zeitpunkt | Anmerkung/ Grund der Verzögerung |
| Anbau Oxygesic – PCA | POD 2 __/__/___ |  |  |
| Entf. Transurethraler DK | POD 3 __/__/___ |  |  |
| Entf. PDK | POD 3 __/__/___ |  |  |
| Entfernung ZVK | POD 7 __/__/___ |  |  |
|  |  |  |  |
|  |  |  |  |
|  |  |  |  |
| **Entlassung** | Geplanter Zeitpunkt | Durchgeführter Zeitpunkt | Anmerkung/ Grund der Verzögerung |
| Vorbereitung Entlassung | POD 6  __/__/___ |  |  |
| Entlassung | POD 7 __/__/___ |  |  |
|  |  |  |  |
| **Mobilisation** | Geplanter Zeitpunkt | Durchgeführter Zeitpunkt | Anmerkung/ Grund der Verzögerung |
| Am Bett |  |  |  |
| Auf den Beinen |  |  |  |
| Selbstmobilisation |  |  |  |
| Erster Stuhlgang |  |  |  |
|  |  |  |  |

Schmerzmittelbedarf**: Analgesieprotokoll**

Bitte Gesamtmenge pro Tag angeben.

| **PDK** | **Präparat/ Einheit** | **POD 0** | **POD 1** | **POD 2** | **POD 3** | **POD** | **POD** |
| --- | --- | --- | --- | --- | --- | --- | --- |
| Präparat: | Naropin ______ % |  |  |  |  |  |  |
|  | Sufentanil [ug/ml] | ______ | ______ | _______ | ______ | _______ | _______ |
| Menge: | Laufzeit [ml/h] |  |  |  |  |  |  |

**Weitere verabreichte Schmerzmittel (Fortsetzung Analgesieprotokoll):**

| **Medikament** | **VAS** | **PCA** |  | | **Novalgin** |  |  |  |  |
| --- | --- | --- | --- | --- | --- | --- | --- | --- | --- |
| Dosierung | VAS Wert | Oxygesic 10mg/ml |  | |  |  |  |  |  |
| Menge/ Tag  POD 0 |  |  |  | |  |  |  |  |  |
| Menge/ Tag  POD 1 |  |  |  | |  |  |  |  |  |
| Menge/ Tag  POD 2 |  |  |  | |  |  |  |  |  |
| Menge/ Tag  POD 3 |  |  |  | |  |  |  |  |  |
| Menge/ Tag  POD 4 |  |  |  | |  |  |  |  |  |
| Menge/ Tag  POD 5 |  |  |  | |  |  |  |  |  |
| Menge/ Tag  POD 6 |  |  |  | |  |  |  |  |  |
| Menge/ Tag  POD 7 |  |  |  | |  |  |  |  |  |
| Menge/ Tag  POD 8 |  |  |  | |  |  |  |  |  |
| Menge/ Tag  POD |  |  |  |  | |  |  |  |  |
| Menge/ Tag  POD |  |  |  |  | |  |  |  |  |
| Menge/ Tag  POD |  |  |  |  | |  |  |  |  |

Weitere Schmerzmittel: __________________________________________________________________

__________________________________________________________________

__________________________________________________________________

__________________________________________________________________

__________________________________________________________________

__________________________________________________________________

| **Zeitpunkt** | **Leukozyten** | **CRP** |
| --- | --- | --- |
| prä-OP |  |  |
| 1. Tag post-OP |  |  |
| 3. Tag post-OP |  |  |
| 4. Tag post-OP |  |  |
| 6. Tag post-OP |  |  |
| 7. Tag post-OP |  |  |
| POD ______ |  |  |
| POD ______ |  |  |
| POD ______ |  |  |

Laborparameter:

**Visite 3: Post – operativer Tag 1 (POD 1): Datum: ___/___/______**

**Verlegung auf Normalstation** ⬜ Ja ⬜ Nein 🡪 Grund in Fast Track Protokoll angeben

**Drainagenentfernung** gemäß SOP:

Abdominelle Drainage entfernt (falls vorhanden) ⬜ Ja ⬜ Nein 🡪 Grund in Fast Track Protokoll angeben

Linksseitige Thoraxdrainage entfernt ⬜ Ja ⬜ Nein 🡪 Grund in Fast Track Protokoll angeben

**Ernährung** gemäß SOP ⬜ Ja ⬜ Nein 🡪 Grund in Fast Track Protokoll angeben

**Schmerztherapie**:

Visuelle Analogskala: ⬜ Ja: Wert _______ ⬜ Nein: Grund _____________________________

PDK: Vorhanden ⬜ Ja 🡪 Laufzeit in Schmerzprotokoll eintragen ⬜ Nein

Zusätzliche Schmerzmedikation ⬜ Ja 🡪 In Schmerzprotokoll eintragen ⬜ Nein

**Mobilisation**: ⬜ Ja 🡪 Form ⬜ Nein

Form der Mobilisation: ________________________________________

**Stuhlgang**: ⬜ Ja ⬜ Nein

**Wundpflege**:

Wundheilungsstörung ⬜ Ja ⬜ Nein

Falls „ja“ bitte weiter mit Komplikationen – Erfassung und

⬜ ASEPSIS Score ausfüllen

⬜ CDC – Einteilung ausfüllen

Notwendigkeit einer speziellen Wundpflege ⬜ Ja ⬜ Nein

Wenn ja, welche: ________________________________________________

**Morbidität:** Auftreten von Komplikationen ⬜ Ja ⬜ Nein

Falls „Ja“ bitte angeben: _____________________________________________________________

_________________________________________________________________________________

**Bei Abweichungen/ Besonderheiten bitte präzisieren**: __________________________________________

_______________________________________________________________________________________

_______________________________________________________________________________________

_______________________________________________________________________________________

**Durchgeführt von:**

**_______________**

**Visite 4: POD 2 Datum: ___/___/______**

**Drainagen – und Katheterentfernung** gemäß SOP:

Entfernung rechte thor. Bülaudrainage ⬜ Ja ⬜ Nein 🡪 Grund in Fast Track Protokoll angeben

**Ernährung**: Gemäß SOP ⬜ Ja ⬜ Nein 🡪 Grund in Fast Track Protokoll angeben

**Schmerztherapie**:

Visuelle Analogskala: ⬜ Ja, Wert: ____ ⬜ Nein, Grund: _______________________

PDK: Vorhanden ⬜ Ja 🡪 Laufzeit in Schmerzprotokoll angeben ⬜ Nein

Oxygesic – PCA: Anbau ⬜ Ja ⬜ Nein 🡪 Grund in Fast Track Protokoll angeben

Zusätzliche Schmerzmedikation ⬜ Ja 🡪 Menge in Schmerzprotokoll angeben ⬜ Nein

**Mobilisation**: ⬜ Ja 🡪 Form ⬜ Nein

Form der Mobilisation: _________________________________________

**Stuhlgang**: ⬜ Ja ⬜ Nein

**Wundpflege**:

. Wundheilungsstörung ⬜ Ja ⬜ Nein

Falls „ja“ bitte weiter mit Komplikationen – Erfassung und

⬜ ASEPSIS Score ausfüllen

. ⬜ CDC – Einteilung ausfüllen

Notwendigkeit einer speziellen Wundpflege ⬜ Ja ⬜ Nein

Wenn ja, welche: ________________________________________________

**Morbidität:** Auftreten von Komplikationen ⬜ Ja ⬜ Nein

Falls „Ja“ bitte angeben: _____________________________________________________________

_________________________________________________________________________________

**Bei Abweichungen/ Besonderheiten bitte präzisieren**: __________________________________________

_______________________________________________________________________________________

_______________________________________________________________________________________

**Durchgeführt von:**

**_______________**

**Visite 5: POD 3 Datum: ___/___/______**

**Drainagen – und Katheterentfernung** gemäß SOP:

Entfernung des transurethralen Dauerkatheters ⬜ Ja ⬜ Nein 🡪 Grund in Fast Track Protokoll angeben

**Ernährung**: Gemäß SOP ⬜ Ja ⬜ Nein 🡪 Grund in Fast Track Protokoll angeben

**Schmerztherapie**:

Visuelle Analogskala: ⬜ Ja, Wert: _____ ⬜ Nein, Grund: ___________________________

Oxygesic – PCA: ⬜ Ja 🡪 Menge in Analgesieprotokoll angeben ⬜ Nein 🡪 Grund in FTP angeben

Zusätzliche Schmerzmedikation nach Analgesie – SOP ⬜ Ja 🡪 Menge Analgesieprotokoll ⬜ Nein 🡪 Grund in FTP angeben

**Mobilisation**: ⬜ Ja 🡪 Form ⬜ Nein

Form der Mobilisation: _________________________________________

**Stuhlgang**: ⬜ Ja ⬜ Nein

**Wundpflege**:

Wundheilungsstörung: ⬜ Ja ⬜ Nein

Falls „ja“ bitte weiter mit Komplikationen – Erfassung und

⬜ ASEPSIS Score ausfüllen

. ⬜ CDC – Einteilung ausfüllen

Notwendigkeit einer speziellen Wundpflege ⬜ Ja ⬜ Nein

. Wenn ja, welche: ________________________________________________

**Morbidität:** Auftreten von Komplikationen ⬜ Ja ⬜ Nein

Falls „Ja“ bitte angeben: _____________________________________________________________

_________________________________________________________________________________

**Bei Abweichungen/ Besonderheiten bitte präzisieren**: __________________________________________

_______________________________________________________________________________________

_______________________________________________________________________________________

**Visite durchgeführt von:**

**____________________**

**Visite 6: POD 4 Datum ___/___/______**

**Ernährung**:

Sondennahrung gemäß SOP ⬜ Ja ⬜ Nein 🡪 Grund in Fast Track Protokoll angeben

**Schmerztherapie**:

Visuelle Analogskala: ⬜ Ja, Wert: _____ ⬜ Nein, Grund: ____________________________

Oxygesic – PCA: ⬜ Ja 🡪 Menge in Analgesieprotokoll ⬜ Nein 🡪 Grund in FTP angeben

Zusätzliche Schmerzmedikation ⬜ Ja 🡪 Menge in Analgesieprotokoll ⬜ Nein

**Mobilisation**: ⬜ Ja 🡪 Form ⬜ Nein

Falls „Ja“: Form der Mobilisation: _________________________________________

**Stuhlgang**: ⬜ Ja ⬜ Nein

**Wundpflege**:

Wundheilungsstörung ⬜ Ja ⬜ Nein

Falls „ja“ bitte weiter mit Komplikationen – Erfassung und

⬜ ASEPSIS Score ausfüllen

. ⬜ CDC – Einteilung ausfüllen

Notwendigkeit einer speziellen Wundpflege ⬜ Ja ⬜ Nein

Wenn ja, welche: ________________________________________________

**Morbidität:** Auftreten von Komplikationen ⬜ Ja ⬜ Nein

Falls „Ja“ bitte angeben: _____________________________________________________________

_________________________________________________________________________________

**Bei Abweichungen/ Besonderheiten bitte präzisieren**: __________________________________________

_______________________________________________________________________________________

_______________________________________________________________________________________

**Visite durchgeführt von:**

**____________________**

**Visite 7: POD 5 Datum ___/___/______**

**Ernährung**:

Beginn des Kostaufbaus (Kostform Stoma I) ⬜ Ja ⬜ Nein 🡪 Grund in Fast Track Protokoll angeben

Entfernung der Magensonde ⬜ Ja ⬜ Nein 🡪 Grund in Fast Track Protokoll angeben

**Entfernung der thorakalen Zieldrainage** ⬜ Ja ⬜ Nein 🡪 Grund in Fast Track Protokoll angeben

**Schmerztherapie**:

Visuelle Analogskala: ⬜ Ja, Wert: _____ ⬜ Nein, Grund: __________________________

Oxygesic – PCA: ⬜ Ja 🡪 Menge in Analgesieprotokoll ⬜ Nein 🡪 Grund in FTP angeben

Zusätzliche Schmerzmedikation ⬜ Ja 🡪 Menge in Analgesieprotokoll ⬜ Nein

**Mobilisation**: ⬜ Nein ⬜ Ja: Form: _________________________________________

**Stuhlgang**: ⬜ Ja ⬜ Nein

**Wundpflege**:

Wundheilungsstörung ⬜ Ja ⬜ Nein

Falls „ja“ bitte weiter mit Komplikationen – Erfassung und

⬜ ASEPSIS Score ausfüllen

. ⬜ CDC – Einteilung ausfüllen

Notwendigkeit einer speziellen Wundpflege ⬜ Ja ⬜ Nein

Wenn ja, welche: ___________________________________________________

**Morbidität:** Auftreten von Komplikationen ⬜ Ja ⬜ Nein

Falls „Ja“ bitte angeben: _____________________________________________________________

_________________________________________________________________________________

**Bei Abweichungen/ Besonderheiten bitte präzisieren**: __________________________________________

_______________________________________________________________________________________

_______________________________________________________________________________________

_______________________________________________________________________________________

**Visite durchgeführt von:**

**____________________**

**Visite 8: POD 6 Datum ___/___/______**

**Ernährung**:

Steigerung des Kostaufbaus (Kostform Stoma II) ⬜ Ja ⬜ Nein 🡪 Grund in Fast Track Protokoll angeben

Ende parenterale Ernährung ⬜ Ja ⬜ Nein 🡪 Grund in Fast Track Protokoll angeben

**Vorbereitung der Entlassung** ⬜ Ja ⬜ Nein 🡪 Grund in Fast Track Protokoll angeben

**Schmerztherapie**:

Visuelle Analogskala: ⬜ Ja, Wert: ____ ⬜ Nein, Grund: ___________________________

Oxygesic – PCA: ⬜ Ja 🡪 Menge in Analgesieprotokoll ⬜ Nein 🡪 Grund in FTP angeben

Zusätzliche Schmerzmedikation nach Analgesie – SOP ⬜ Ja 🡪 Menge in Analgesieprotokoll ⬜ Nein

**Mobilisation**: ⬜ Nein ⬜ Ja, Form: _________________________________

**Stuhlgang**: ⬜ Ja ⬜ Nein

**Wundpflege**:

Wundheilungsstörung ⬜ Ja ⬜ Nein

Falls „ja“ bitte weiter mit Komplikationen – Erfassung und

⬜ ASEPSIS Score ausfüllen

. ⬜ CDC – Einteilung ausfüllen

Notwendigkeit einer speziellen Wundpflege ⬜ Ja ⬜ Nein

Wenn ja, welche: ________________________________________________

**Morbidität:** Auftreten von Komplikationen ⬜ Ja ⬜ Nein

Falls „Ja“ bitte angeben: _____________________________________________________________

_________________________________________________________________________________

**Bei Abweichungen/ Besonderheiten bitte präzisieren**: __________________________________________

_______________________________________________________________________________________

_______________________________________________________________________________________

**Visite durchgeführt von:**

**____________________**

**Visite 9: POD 7** Datum: _____/_____/___________

**Ernährung:**

Kostform: Stoma III ⬜ Ja ⬜ Nein 🡪 Grund in Fast Track Protokoll angeben

**Durchführung einer Ernährungsberatung** ⬜ Ja ⬜ Nein 🡪 Grund in Fast Track Protokoll angeben

**Entfernung des Zentralen Venenkatheters** ⬜ Ja ⬜ Nein 🡪 Grund in Fast Track Protokoll angeben

**SOP:** Vervollständigung des Fast Track Protokolls ⬜ Ja ⬜ Nein 🡪 Grund:

(zB kein Drainagenzug) __________________________________________________________

**Fragebögen:**

Quality of Recovery 15 Fragebogen ausgefüllt ⬜ Ja ⬜ Nein 🡪 Grund:

____________________________________________________________________

**Morbidität:**

Auftreten von Komplikationen ⬜ Ja ⬜ Nein

Falls „Ja“ bitte angeben und im Teil „Komplikationen“ ergänzen.
________________________________________________________________________________
________________________________________________________________________________

**Drainagen/ Katheter**:

Entfernung von Drainagen oder Katheter ⬜ Ja 🡪 in Übersichtstabelle eintragen ⬜ Nein

**Bei Abweichungen/ Besonderheiten bitte präzisieren**: ___________________________

________________________________________________________________________

________________________________________________________________________

________________________________________________________________________

________________________________________________________________________

**Visite durchgeführt von: _______________________**

QoR-15; Quality of Recovery 15

**
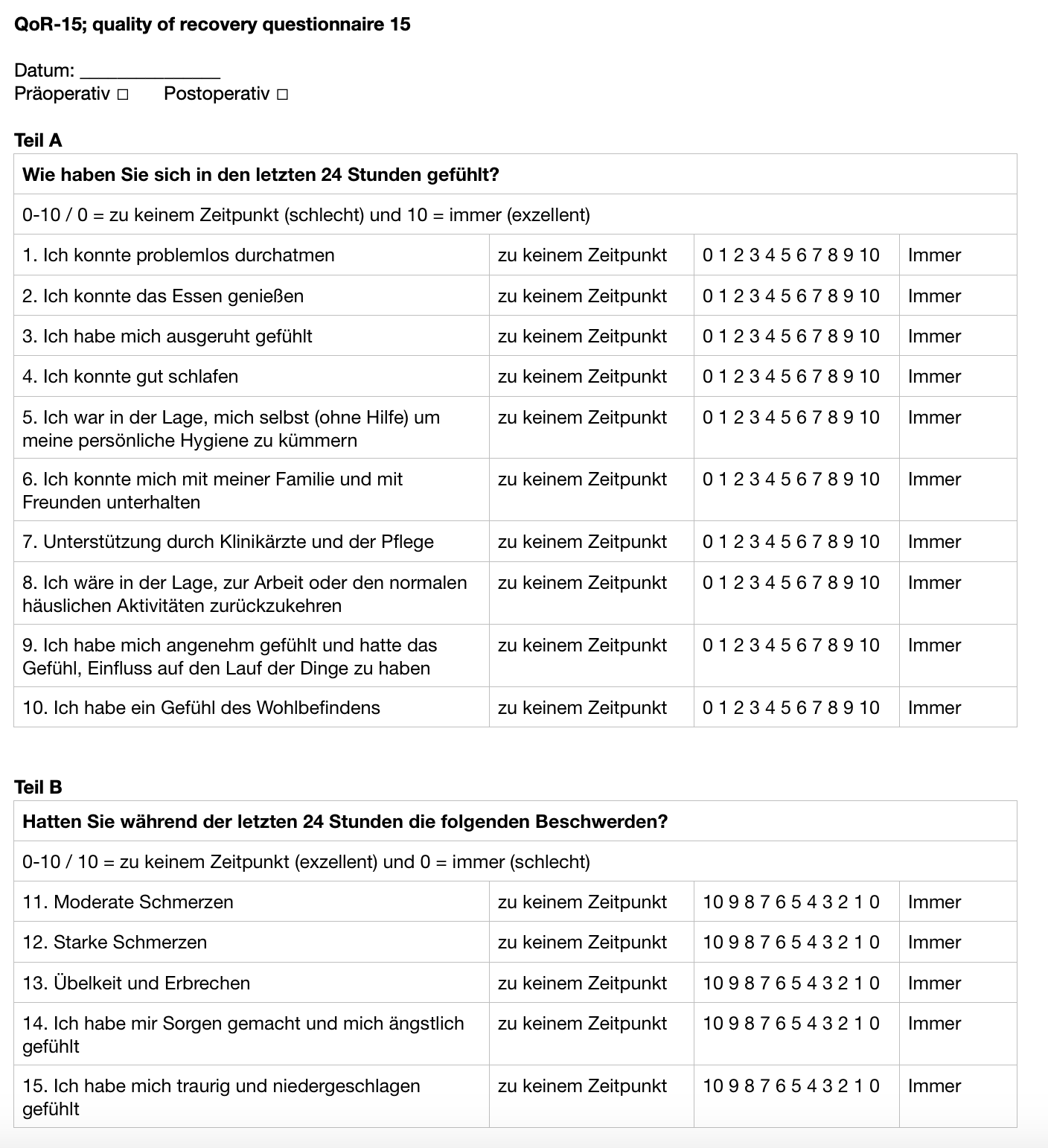
**

**Visite ____:** Datum: _____/_____/___________

**SOP:** Vervollständigung des Fast Track Protokolls ⬜ Ja ⬜ Nein 🡪 Grund

**Morbidität:**

Auftreten von Komplikationen ⬜ Ja ⬜ Nein

Falls „Ja“ bitte weiter mit Komplikationen Erfassung bis inkl. POD 30

**Drainagen/ Katheter**:

Entfernung von Drainagen oder Katheter ⬜ Ja ⬜ Nein

Falls „Ja“: welche: ______________________________________________

**Bei Abweichungen/ Besonderheiten bitte präzisieren**: ___________________________

________________________________________________________________________

________________________________________________________________________

________________________________________________________________________

________________________________________________________________________

________________________________________________________________________

**Visite durchgeführt von: ________________**

**Visite 10: Entlass-Visite Datum: _____/_____/___________**

**Kontaktmöglichkeit**: ______________________________________________

**Krankenhausaufenthalt**:

| Länge des Aufenthaltes auf Intensivstation | Tage: |
| --- | --- |
| Länge des Intermediate Care Aufenthaltes | Tage: |
| Dauer der Lungenbeatmung | Tage: |
| Länge des Gesamtaufenthaltes im Krankenhaus ab OP (= Tag 1) | Tage: |

**Histopathologische Daten**:
Tumorart:

⬜ Adenokarzinom ⬜ Plattenepithelkarzinom

⬜ NET ⬜ Anderes: _________________________

TNM-Klassifikation: T: __________N: _____________________ M: ___________

Staging: ______________________________, Grading: ________________________________

R0-Resektion: ⬜ ja ⬜ Nein, Lage Residualtumor: __________________________________

**Morbidität/ Mortalität**:

Auftreten von Komplikationen innerhalb der ersten 30 POD bzw. während des KH Aufenthaltes?

⬜ nein ⬜ ja 🡪 Weiter mit „Komplikationen –Erfassung bis inkl. POD 30“

**Fragebögen:**

QoR-15 (Quality of Recovery 15) ⬜ _______________

Lebensqualitätserfassung: SF-36 ⬜ _______________

EORTC QLQ-C30 ⬜ _______________

EORTC QLQ- OES18 ⬜ _______________

**Entlassung am ___/___/______ (Tag/ Mt/ Jahr)**

**⬜ Nach Hause**

**⬜ In Reha: wohin ____________________, warum____________________________**

**_______________________________________________________________**

**⬜ In andere Klinik: wohin ____________________, warum______________________**

**_______________________________________________________________**

QoR-15; Quality of Recovery 15

**
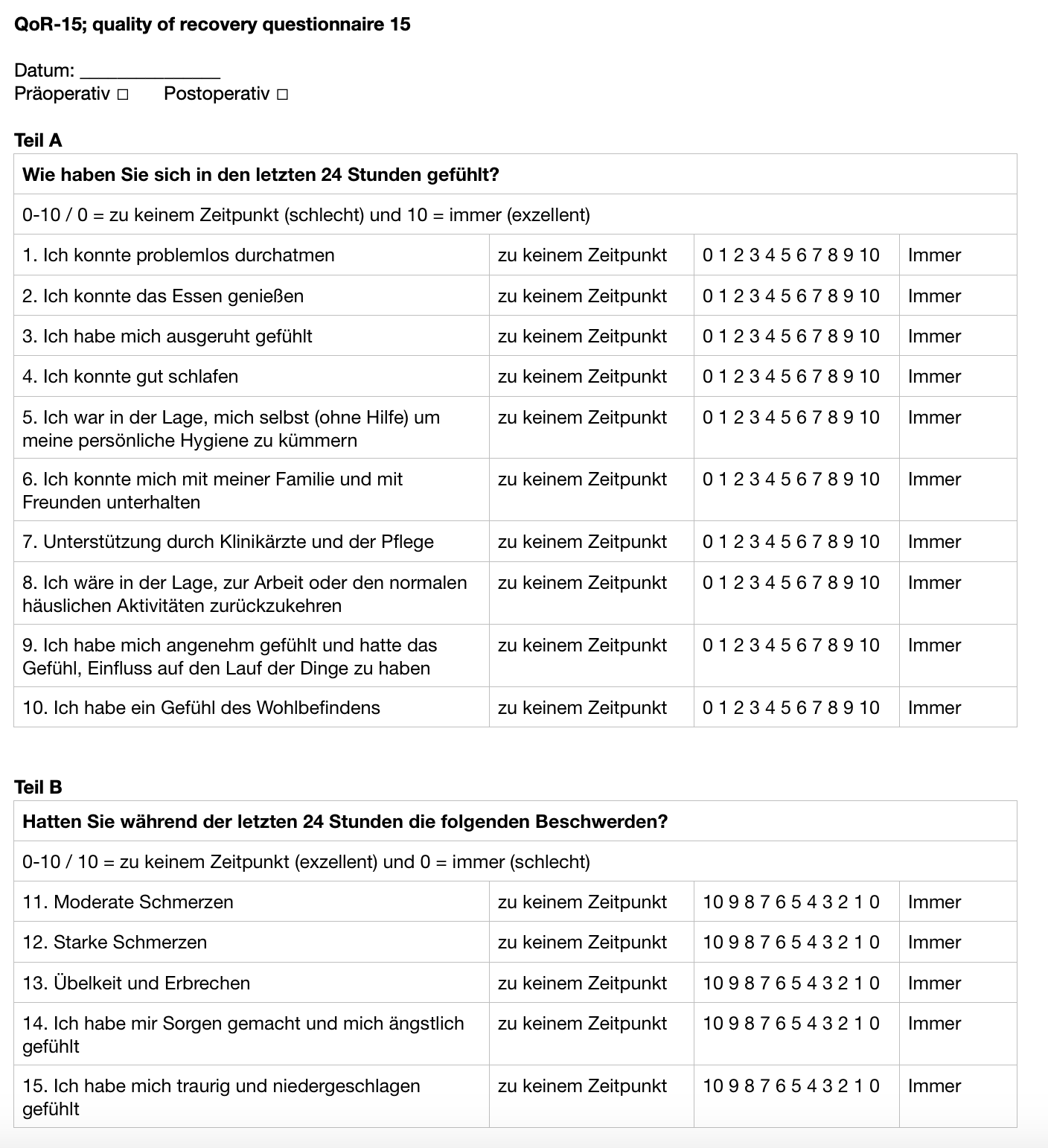
**

SF-36 Fragebogen: (Siehe vorgefertigter Ausdruck)

EORTC QLQ-C30


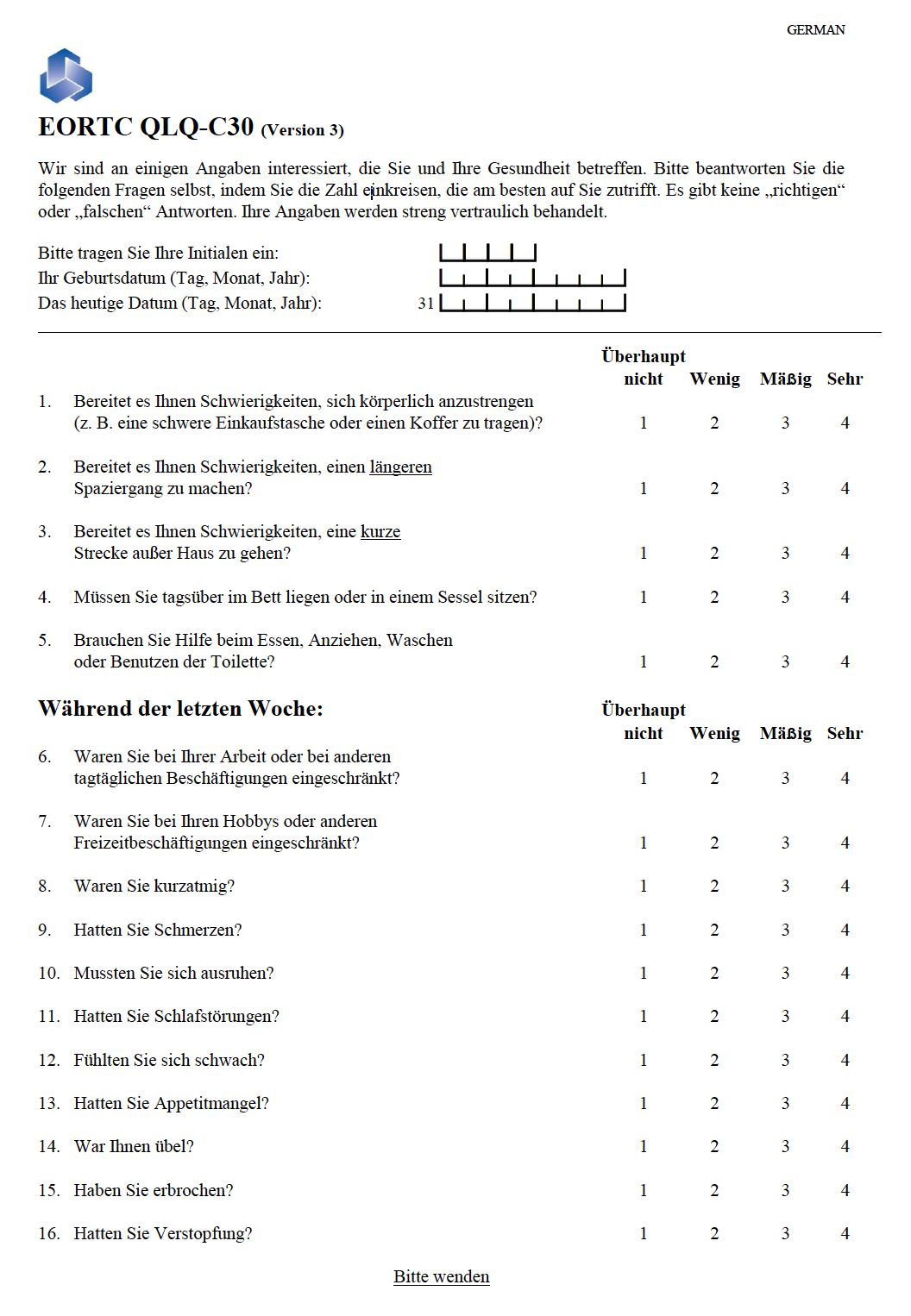


**
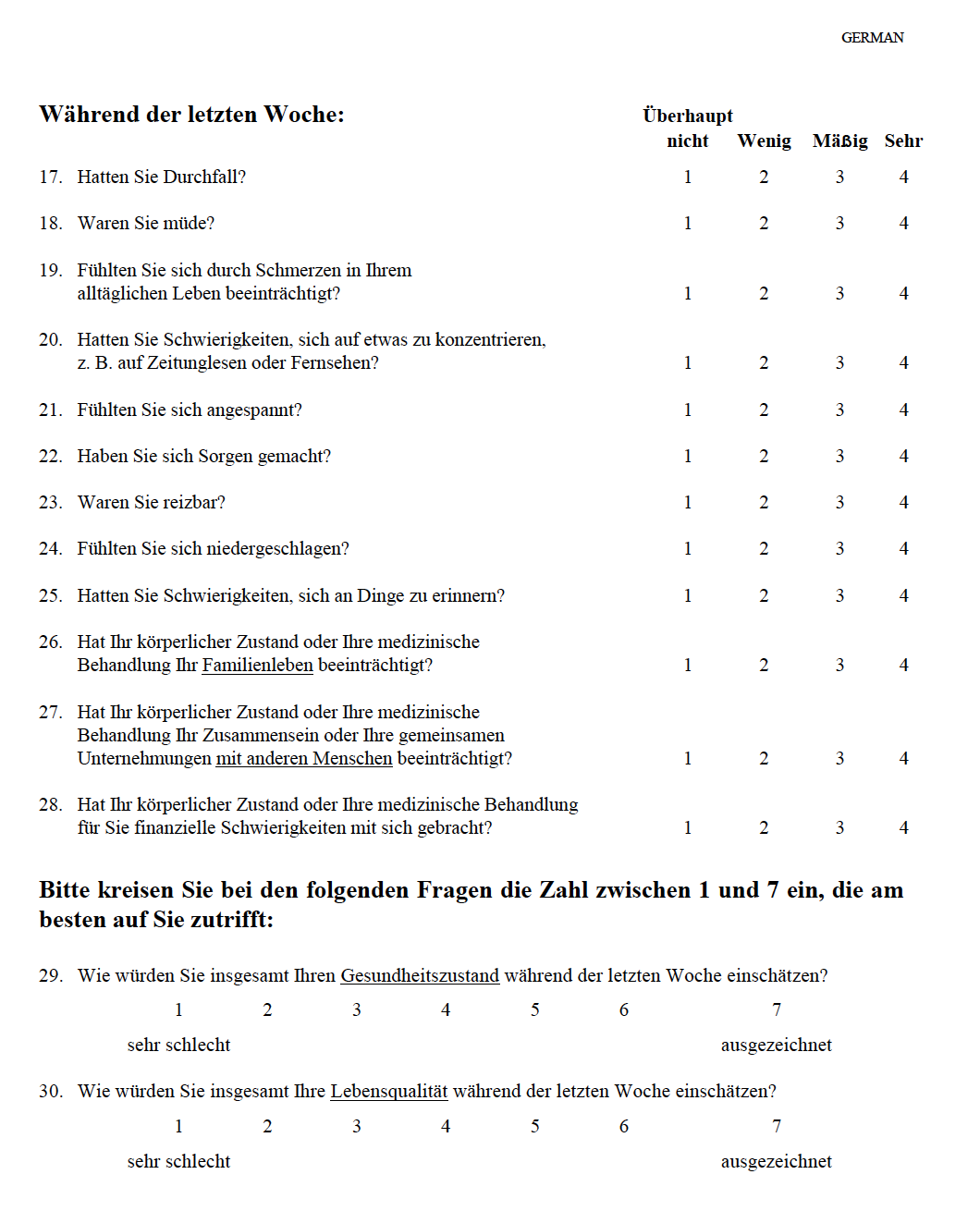
**

EORTC QLQ-OES18

**
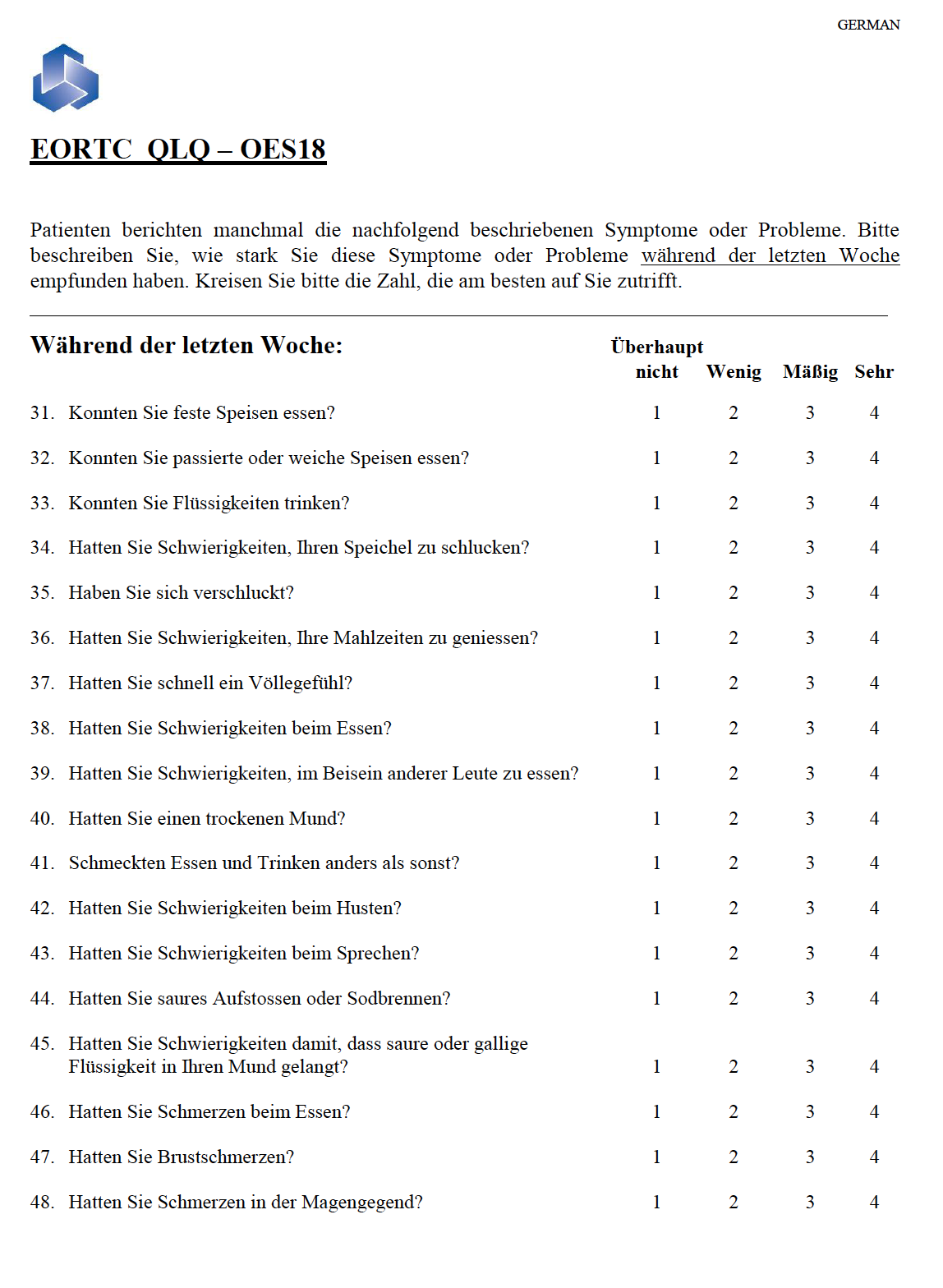
**

Summe: Datum:

**Überblick über bisher stattgefundene Komplikationen bis inkl. POD 30**:

| Komplikation | Datum des Auftretens | Folge/ Intervention | Clav.Dindo Grad | Datum und Name Eintragender |
| --- | --- | --- | --- | --- |
|  |  |  |  |  |
|  |  |  |  |  |
|  |  |  |  |  |
|  |  |  |  |  |
|  |  |  |  |  |
|  |  |  |  |  |
|  |  |  |  |  |
|  |  |  |  |  |
|  |  |  |  |  |
|  |  |  |  |  |
|  |  |  |  |  |

**Komplikationen – Erfassung:
Erfassung aller Komplikationen bis inkl. POD 30:** (später aufgetretene Komplikationen bitte beim Komplikationsteil „Komplikationen nach POD 30“ eintragen)

Anastomotic leak: (nach ECCG, Anhang 9.) ⬜ ja ⬜ nein

Zeitpunkt des Auftretens: POD _____ oder Datum: ____/____/________

⬜ Type I ⬜ Type II ⬜ Type III

Wenn ja, Begründung angeben (inkl. postop. Tag, Intervention)

_________________________________________________________________

_________________________________________________________________

_________________________________________________________________

_________________________________________________________________

_________________________________________________________________

Endgültige Beurteilung:

Nach Clavien Dindo: I O II O IIIa O IIIb O IVa O IVb O V O

Conduit Necrosis: (nach ECCG, Anhang 9.) ⬜ ja ⬜ nein

Zeitpunkt des Auftretens: POD _____ oder Datum: ____/____/________

⬜ Type I ⬜ Type II ⬜ Type III

Behandlung: _____________________________________________________

Wenn ja, Begründung angeben (inkl. postop. Tag, Intervention)

_________________________________________________________________

_________________________________________________________________

_________________________________________________________________

_________________________________________________________________

_________________________________________________________________

Endgültige Beurteilung:

Nach Clavien Dindo: I O II O IIIa O IIIb O IVa O IVb O V O

Chyle leak: (nach ECCG) ⬜ ja ⬜ nein

Zeitpunkt des Auftretens: POD _____ oder Datum: ____/____/________

⬜ Type I ⬜ Type II ⬜ Type III

Severity Level: ⬜ A ⬜ B

Wenn ja, Begründung angeben (inkl. postop. Tag, Intervention)

_________________________________________________________________

_________________________________________________________________

_________________________________________________________________

_________________________________________________________________

_________________________________________________________________

Endgültige Beurteilung:

Nach Clavien Dindo: I O II O IIIa O IIIb O IVa O IVb O V O

Vocal cord injury/ Palsy: (nach ECCG) ⬜ ja ⬜ nein

Zeitpunkt des Auftretens: POD _____ oder Datum: ____/____/________

⬜ Type I ⬜ Type II ⬜ Type III

Severity Level: ⬜ Unilateral ⬜ Bilateral

Wenn ja, Begründung angeben (inkl. postop. Tag, Intervention)

_________________________________________________________________

_________________________________________________________________

_________________________________________________________________

_________________________________________________________________

_________________________________________________________________

Endgültige Beurteilung:

Nach Clavien Dindo: I O II O IIIa O IIIb O IVa O IVb O V O

Surgical Site Infection: (CDC, Anhang 6.) ⬜ ja ⬜ nein

Zeitpunkt des Auftretens: POD _____ oder Datum: ____/____/________

Grad nach CDC: ⬜ superficial ⬜ deep ⬜ organ/ space

ASEPSIS Score: (Siehe Anhang 6. ASEPSIS – Score)

⬜ Satisfactory healing ⬜ Disturbance of healing

⬜ Minor wound infection ⬜ Moderate Wound Infection

⬜ Severe Wound Infection

Länge der Wunde [cm]: ________

Wenn ja, Begründung angeben (inkl. postop. Tag, Intervention)

_________________________________________________________________

_________________________________________________________________

_________________________________________________________________

_________________________________________________________________

_________________________________________________________________

Endgültige Beurteilung:

Nach Clavien Dindo: I O II O IIIa O IIIb O IVa O IVb O V O

Weitere Komplikationen: ⬜ ja ⬜ nein

Zeitpunkt des Auftretens: POD _____ oder Datum: ____/____/________

Wenn ja, Begründung angeben (inkl. postop. Tag, Intervention)

_________________________________________________________________

_________________________________________________________________

_________________________________________________________________

_________________________________________________________________

_________________________________________________________________

Endgültige Beurteilung

Nach Clavien Dindo: I O II O IIIa O IIIb O IVa O IVb O V O

Weitere Komplikationen: (Siehe Anhang, 9.) ⬜ ja ⬜ nein

Zeitpunkt des Auftretens: POD _____ oder Datum: ____/____/________

Wenn ja, Begründung angeben (inkl. postop. Tag, Intervention)

_________________________________________________________________

_________________________________________________________________

_________________________________________________________________

_________________________________________________________________

_________________________________________________________________

Endgültige Beurteilung

Nach Clavien Dindo: I O II O IIIa O IIIb O IVa O IVb O V O

Weitere Komplikationen: (Siehe Anhang, 9.) ⬜ ja ⬜ nein

Zeitpunkt des Auftretens: POD _____ oder Datum: ____/____/________

Wenn ja, Begründung angeben (inkl. postop. Tag, Intervention)

_________________________________________________________________

_________________________________________________________________

_________________________________________________________________

_________________________________________________________________

_________________________________________________________________

Endgültige Beurteilung:

Nach Clavien Dindo: I O II O IIIa O IIIb O IVa O IVb O V O

Weitere Komplikationen: ⬜ ja ⬜ nein

Zeitpunkt des Auftretens: POD _____ oder Datum: ____/____/________

Wenn ja, Begründung angeben (inkl. postop. Tag, Intervention)

_________________________________________________________________

_________________________________________________________________

_________________________________________________________________

_________________________________________________________________

_________________________________________________________________

Endgültige Beurteilung:

Nach Clavien Dindo: I O II O IIIa O IIIb O IVa O IVb O V O

Interventionen: (bitte weitere nicht – erfasste Interventionen angeben)

Intervention: ⬜ ja ⬜ nein

Falls „ja“ bitte angeben: Zeitpunkt: POD _____ oder Datum: ____/____/________

(Grund, Art)

_____________________________________________________________

_____________________________________________________________

_____________________________________________________________

_____________________________________________________________

Antibiotikatherapie ⬜ ja ⬜ nein

Falls „ja“ bitte angeben: Zeitpunkt: POD _____ oder Datum: ____/____/________,

(Grund, Art)

_____________________________________________________________

_____________________________________________________________

_____________________________________________________________

_____________________________________________________________

Weitere: ⬜ ja ⬜ nein

Falls „ja“ bitte angeben: Zeitpunkt: POD _____ oder Datum: ____/____/________,

(Grund, Art)

_____________________________________________________________

_____________________________________________________________

_____________________________________________________________

_____________________________________________________________

| Ich bestätige nach bestem Wissen und Gewissen, dass die obigen Angaben vollständig, zutreffend und korrekt sind. | |
| --- | --- |
|  |  |
| Name des Prüfarztes | Unterschrift |

**Visite 11: 1. Follow-Up (30 Tage) Datum _____/_____/___________**

⬜ Ja ⬜ Nein

Falls “nein”: Grund angeben

⬜ Rücktritt des Patienten von der Studie ⬜ Patient verstorben
Datum: ___/___/_______

⬜ Patient nicht mehr erreichbar ⬜ Anderer Grund: _________________

**Gewicht:** [kg]: ____________

**Symptomerfassung:**

Reflux: ⬜ Ja ⬜ Nein Medikation: _____________________________

Dysphagie: ⬜ Ja ⬜ Nein Medikation: _____________________________

**Fragebögen:**

Lebensqualitätserfassung (SF-36) ⬜ _______________

EORTC QLQ-C30 ⬜ _______________

EORTC QLQ- OES18 ⬜ _______________

Comprehensive Complication Index: CCI 30 Tage ⬜ _______________

QoR – 15 ⬜ _______________

Rückkehr zur Arbeit/ normalen (häuslichen) Aktivitäten: ____/____/_______

**Lokalrezidiv oder Progressive Disease**:

⬜ Ja ⬜ Nein, Beschreibung: _________________________________________

**Adjuvante Therapie**:

Wirkstoff (Dosis)__________________________ Zyklen: ____________________

Wirkstoff (Dosis)__________________________ Zyklen: ____________________

Wirkstoff (Dosis)__________________________ Zyklen: ____________________

**Morbidität/ Mortalität (< 30 Tage)**

⬜ nein ⬜ ja 🡪 weiter mit „Komplikationen Erfassung bis inkl. POD 30“

## **Name und Unterschrift des Prüfers**:

**Überblick über bisher stattgefundene Komplikationen bis inkl. POD 30**:

| Komplikation | Datum des Auftretens | Folge/ Intervention | Clav.Dindo Grad | Datum und Name Eintragender |
| --- | --- | --- | --- | --- |
|  |  |  |  |  |
|  |  |  |  |  |
|  |  |  |  |  |
|  |  |  |  |  |
|  |  |  |  |  |
|  |  |  |  |  |
|  |  |  |  |  |
|  |  |  |  |  |
|  |  |  |  |  |
|  |  |  |  |  |
|  |  |  |  |  |

**Komplikationen – Erfassung:
Erfassung aller Komplikationen bis inkl. POD 30:** (später aufgetretene Komplikationen bitte beim Komplikationsteil „Komplikationen nach POD 30“ eintragen)

Anastomotic leak: (nach ECCG, Anhang 9.) ⬜ ja ⬜ nein

Zeitpunkt des Auftretens: POD _____ oder Datum: ____/____/________

⬜ Type I ⬜ Type II ⬜ Type III

Wenn ja, Begründung angeben (inkl. postop. Tag, Intervention)

_________________________________________________________________

_________________________________________________________________

_________________________________________________________________

_________________________________________________________________

_________________________________________________________________

Endgültige Beurteilung:

Nach Clavien Dindo: I O II O IIIa O IIIb O IVa O IVb O V O

Conduit Necrosis: (nach ECCG, Anhang 9.) ⬜ ja ⬜ nein

Zeitpunkt des Auftretens: POD _____ oder Datum: ____/____/________

⬜ Type I ⬜ Type II ⬜ Type III

Behandlung: _____________________________________________________

Wenn ja, Begründung angeben (inkl. postop. Tag, Intervention)

_________________________________________________________________

_________________________________________________________________

_________________________________________________________________

_________________________________________________________________

_________________________________________________________________

Endgültige Beurteilung:

Nach Clavien Dindo: I O II O IIIa O IIIb O IVa O IVb O V O

Chyle leak: (nach ECCG) ⬜ ja ⬜ nein

Zeitpunkt des Auftretens: POD _____ oder Datum: ____/____/________

⬜ Type I ⬜ Type II ⬜ Type III

Severity Level: ⬜ A ⬜ B

Wenn ja, Begründung angeben (inkl. postop. Tag, Intervention)

_________________________________________________________________

_________________________________________________________________

_________________________________________________________________

_________________________________________________________________

_________________________________________________________________

Endgültige Beurteilung:

Nach Clavien Dindo: I O II O IIIa O IIIb O IVa O IVb O V O

Vocal cord injury/ Palsy: (nach ECCG) ⬜ ja ⬜ nein

Zeitpunkt des Auftretens: POD _____ oder Datum: ____/____/________

⬜ Type I ⬜ Type II ⬜ Type III

Severity Level: ⬜ Unilateral ⬜ Bilateral

Wenn ja, Begründung angeben (inkl. postop. Tag, Intervention)

_________________________________________________________________

_________________________________________________________________

_________________________________________________________________

_________________________________________________________________

_________________________________________________________________

Endgültige Beurteilung:

Nach Clavien Dindo: I O II O IIIa O IIIb O IVa O IVb O V O

Surgical Site Infection: (CDC, Anhang 6.) ⬜ ja ⬜ nein

Zeitpunkt des Auftretens: POD _____ oder Datum: ____/____/________

Grad nach CDC: ⬜ superficial ⬜ deep ⬜ organ/ space

ASEPSIS Score: (Siehe Anhang 6. ASEPSIS – Score)

⬜ Satisfactory healing ⬜ Disturbance of healing

⬜ Minor wound infection ⬜ Moderate Wound Infection

⬜ Severe Wound Infection

Länge der Wunde [cm]: ________

Wenn ja, Begründung angeben (inkl. postop. Tag, Intervention)

_________________________________________________________________

_________________________________________________________________

_________________________________________________________________

_________________________________________________________________

_________________________________________________________________

Endgültige Beurteilung:

Nach Clavien Dindo: I O II O IIIa O IIIb O IVa O IVb O V O

Weitere Komplikationen: ⬜ ja ⬜ nein

Zeitpunkt des Auftretens: POD _____ oder Datum: ____/____/________

Wenn ja, Begründung angeben (inkl. postop. Tag, Intervention)

_________________________________________________________________

_________________________________________________________________

_________________________________________________________________

_________________________________________________________________

_________________________________________________________________

Endgültige Beurteilung

Nach Clavien Dindo: I O II O IIIa O IIIb O IVa O IVb O V O

Weitere Komplikationen: (Siehe Anhang, 9.) ⬜ ja ⬜ nein

Zeitpunkt des Auftretens: POD _____ oder Datum: ____/____/________

Wenn ja, Begründung angeben (inkl. postop. Tag, Intervention)

_________________________________________________________________

_________________________________________________________________

_________________________________________________________________

_________________________________________________________________

_________________________________________________________________

Endgültige Beurteilung

Nach Clavien Dindo: I O II O IIIa O IIIb O IVa O IVb O V O

Weitere Komplikationen: (Siehe Anhang, 9.) ⬜ ja ⬜ nein

Zeitpunkt des Auftretens: POD _____ oder Datum: ____/____/________

Wenn ja, Begründung angeben (inkl. postop. Tag, Intervention)

_________________________________________________________________

_________________________________________________________________

_________________________________________________________________

_________________________________________________________________

_________________________________________________________________

Endgültige Beurteilung:

Nach Clavien Dindo: I O II O IIIa O IIIb O IVa O IVb O V O

Weitere Komplikationen: ⬜ ja ⬜ nein

Zeitpunkt des Auftretens: POD _____ oder Datum: ____/____/________

Wenn ja, Begründung angeben (inkl. postop. Tag, Intervention)

_________________________________________________________________

_________________________________________________________________

_________________________________________________________________

_________________________________________________________________

_________________________________________________________________

Endgültige Beurteilung:

Nach Clavien Dindo: I O II O IIIa O IIIb O IVa O IVb O V O

Interventionen: (bitte weitere nicht – erfasste Interventionen angeben)

Intervention: ⬜ ja ⬜ nein

Falls „ja“ bitte angeben: Zeitpunkt: POD _____ oder Datum: ____/____/________

(Grund, Art)

_____________________________________________________________

_____________________________________________________________

_____________________________________________________________

_____________________________________________________________

Antibiotikatherapie ⬜ ja ⬜ nein

Falls „ja“ bitte angeben: Zeitpunkt: POD _____ oder Datum: ____/____/________,

(Grund, Art)

_____________________________________________________________

_____________________________________________________________

_____________________________________________________________

_____________________________________________________________

Weitere: ⬜ ja ⬜ nein

Falls „ja“ bitte angeben: Zeitpunkt: POD _____ oder Datum: ____/____/________,

(Grund, Art)

_____________________________________________________________

_____________________________________________________________

_____________________________________________________________

_____________________________________________________________

| Ich bestätige nach bestem Wissen und Gewissen, dass die obigen Angaben vollständig, zutreffend und korrekt sind. | |
| --- | --- |
|  |  |
| Name des Prüfarztes | Unterschrift |

SF-36: (siehe vorgefertigter Ausdruck)

EORTC QLQ-C30


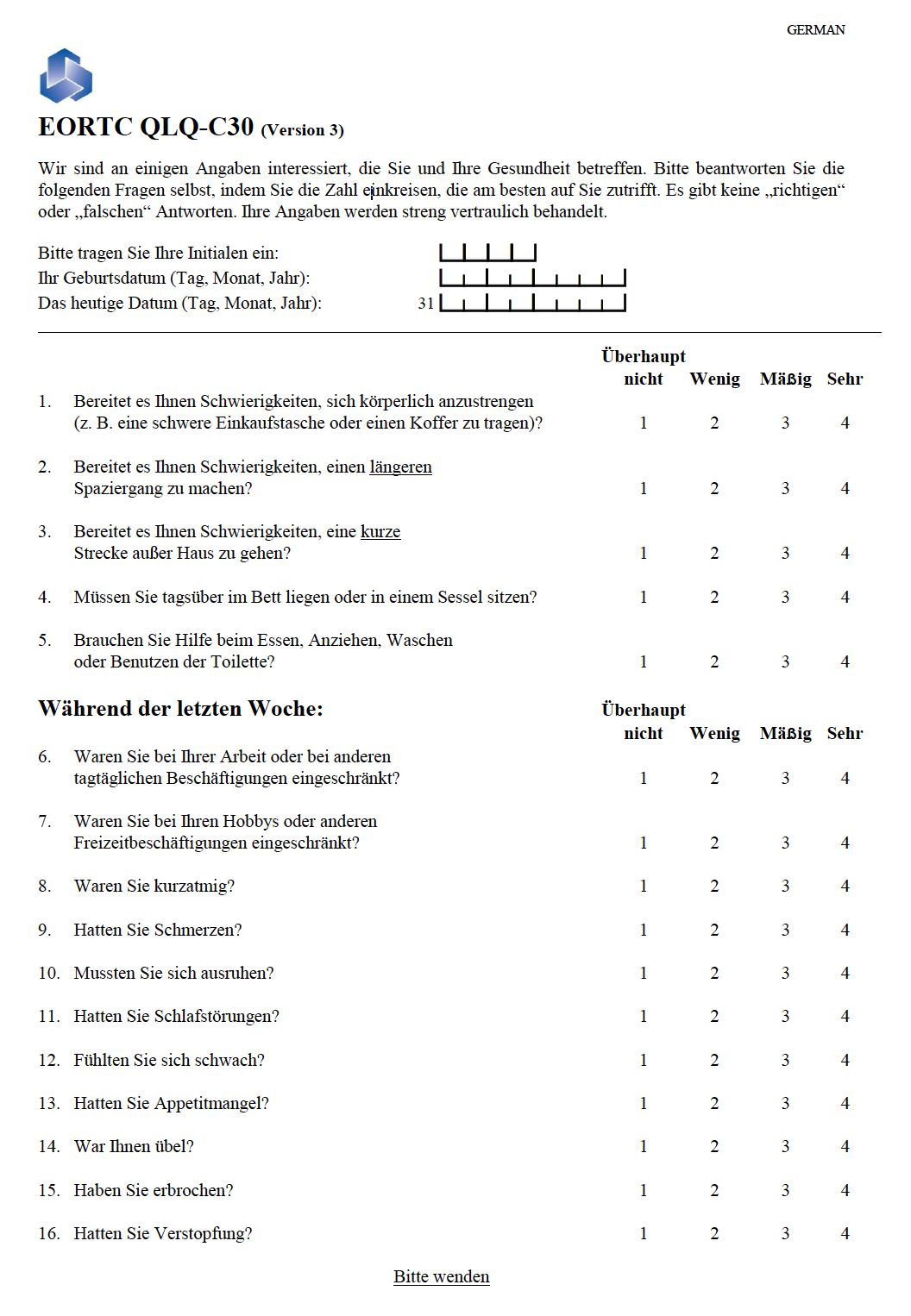


**
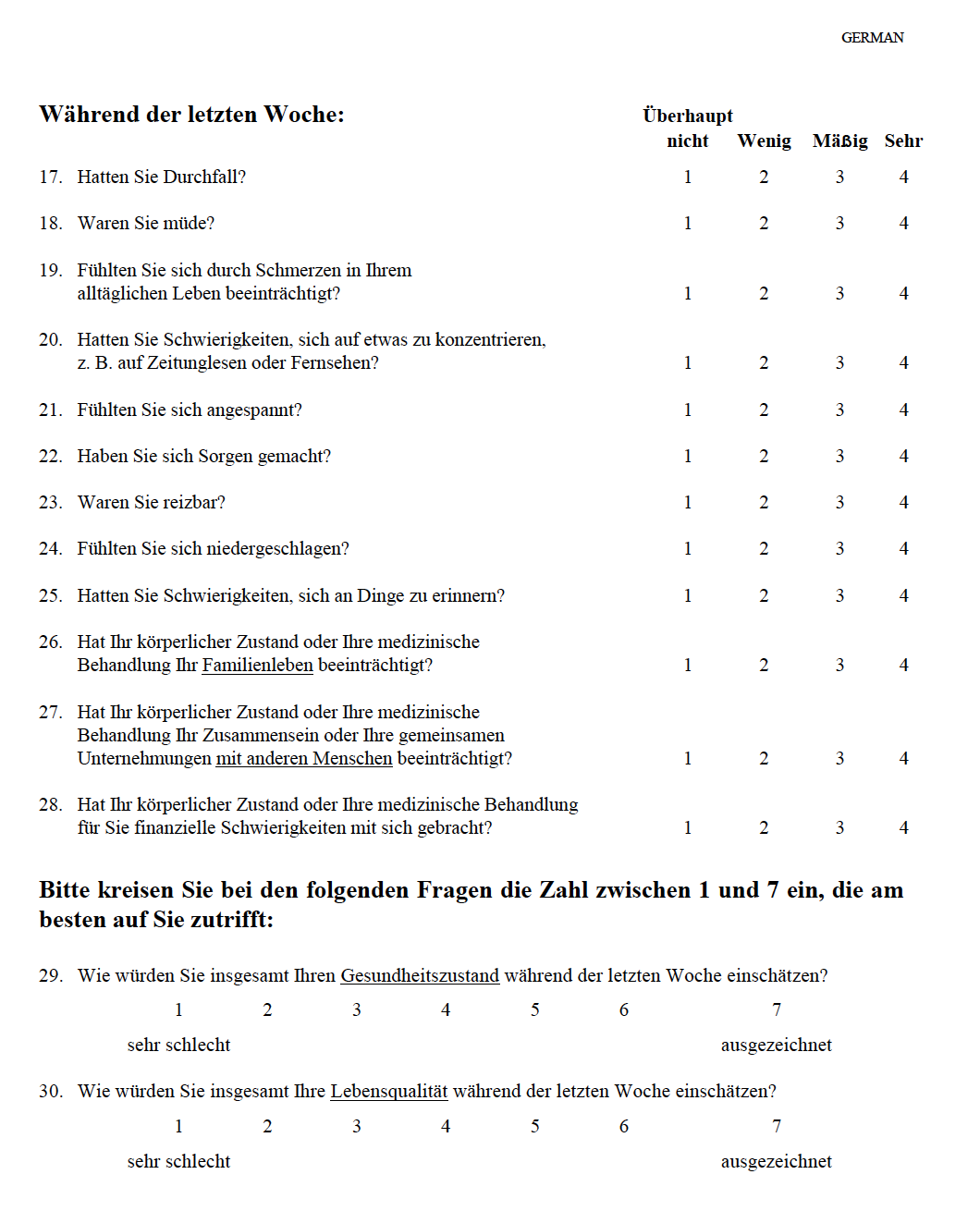
**

Summe: Datum:

EORTC QLQ-OES18

**
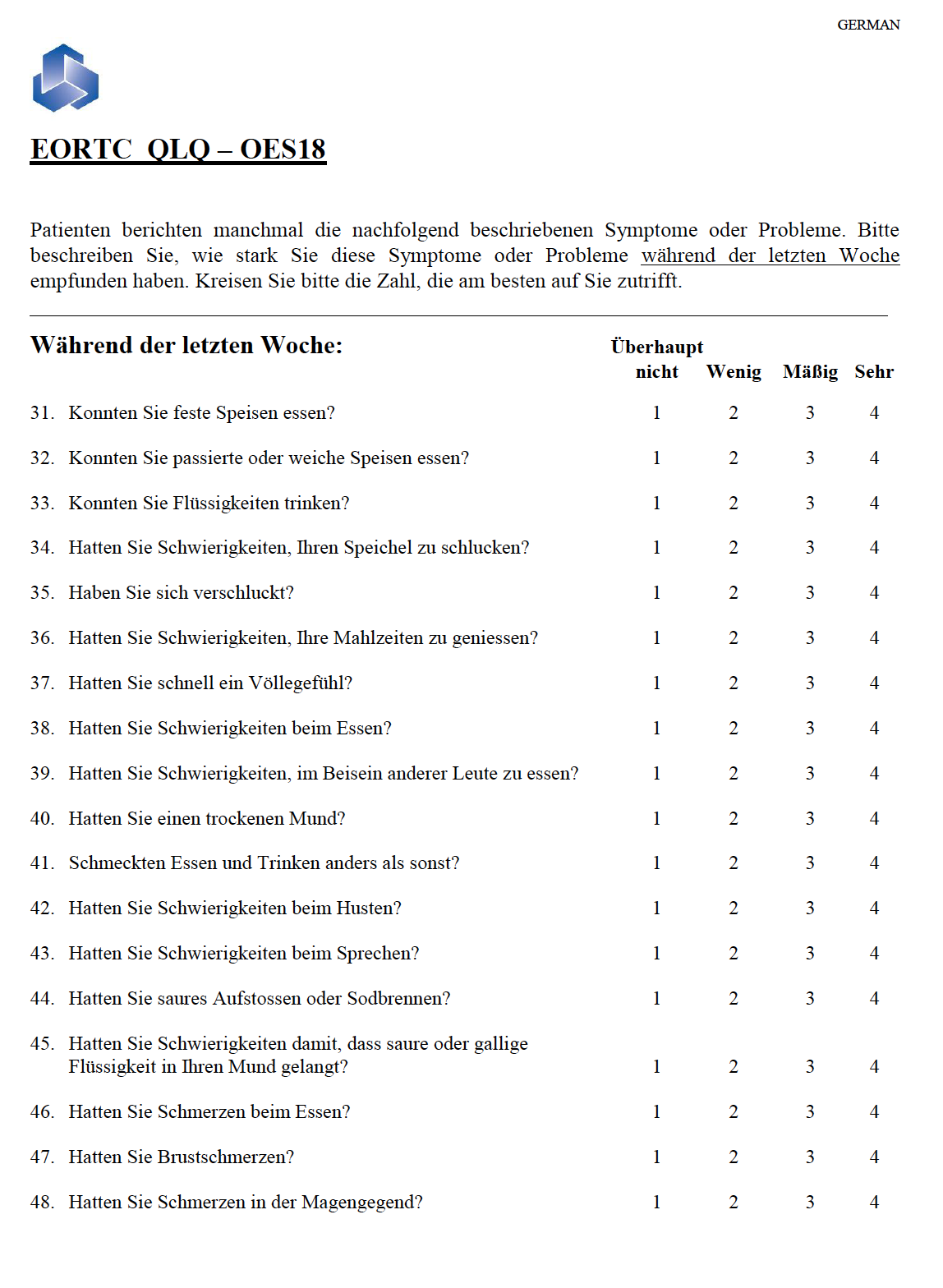
**

Summe: Datum:

QoR-15; Quality of Recovery 15

**
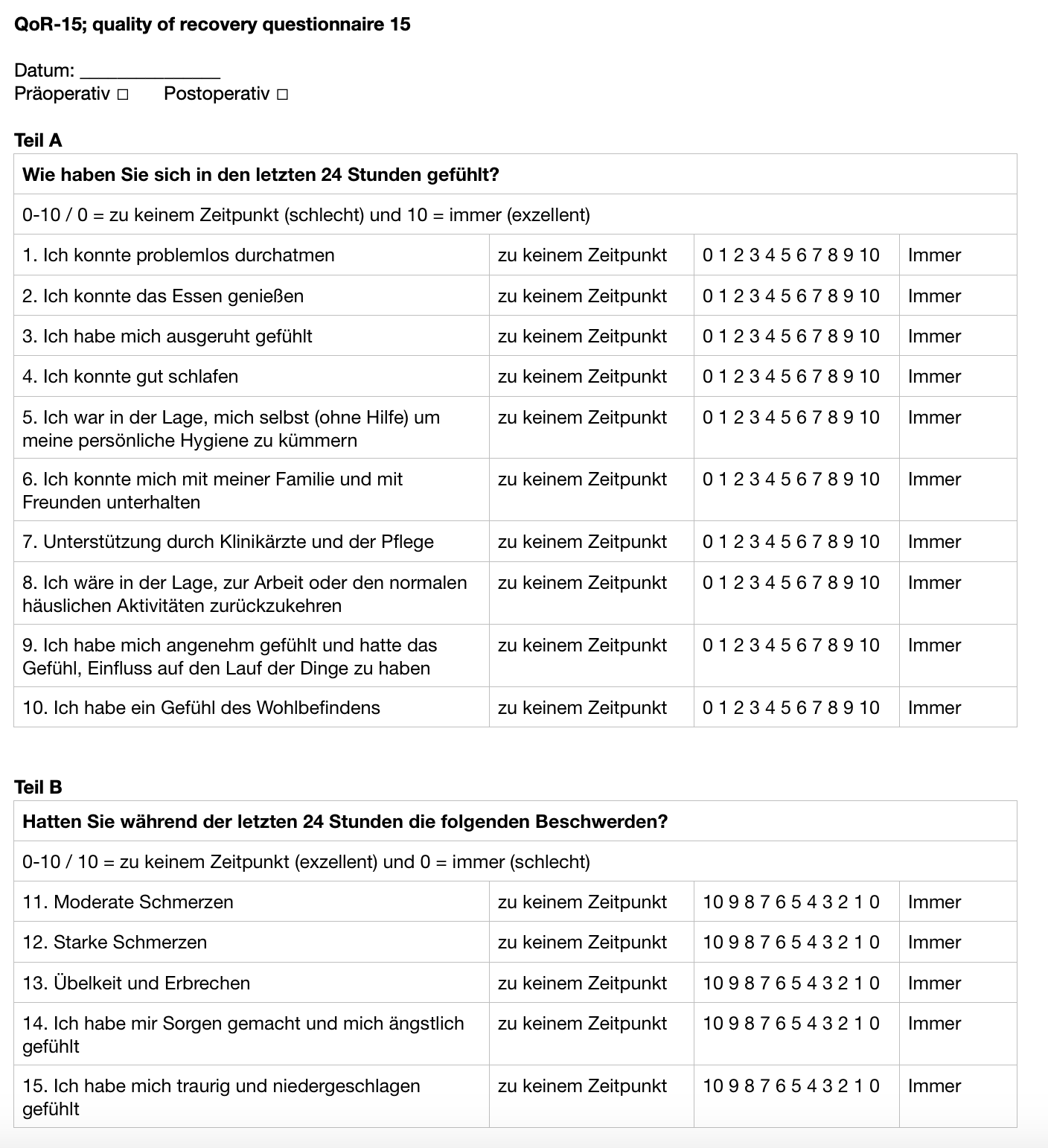
**

Summe Teil A: Summe Teil B:

Summe Gesamt: Datum:

# Visite 12: 2. Follow-Up (3 Monate) Datum: ____/_____/_____________

⬜ Ja ⬜ Nein 🡪 Grund angeben:

⬜ Rücktritt des Patienten von der Studie ⬜ Patient verstorben
Datum: ___/___/______

⬜ Patient nicht mehr erreichbar ⬜ Anderer Grund: _________________

**Gewicht** [kg]: ____________

**Symptomerfassung:**

Reflux: ⬜ Ja ⬜ Nein Medikation: _____________________________

Dysphagie: ⬜ Ja ⬜ Nein Medikation: _____________________________

**Fragebögen:**

Lebensqualitätserfassung (SF-36) ⬜ _______________

EORTC QLQ-C30 ⬜ _______________

EORTC QLQ- OES18 ⬜ _______________

Comprehensive Complication Index: CCI 90 Tage ⬜ _______________

QoR – 15: ⬜ _______________

Rückkehr zur Arbeit/ normalen (häuslichen) Aktivitäten: ____/____/_______

**Lokalrezidiv oder Progressive Disease**:

⬜ Ja ⬜ Nein, Beschreibung: _________________________________________

**Adjuvante Therapie**:

Wirkstoff (Dosis)__________________________ Zyklen: ____________________

Wirkstoff (Dosis)__________________________ Zyklen: ____________________

Wirkstoff (Dosis)__________________________ Zyklen: ____________________

**Morbidität/ Mortalität (> 30 Tage)**

⬜ nein ⬜ ja 🡪 weiter mit „Komplikationen Erfassung nach POD 30“

## **Name und Unterschrift des Prüfers**:

**Überblick über bisher stattgefundene Komplikationen nach POD 30**:

| Komplikation | Datum des Auftretens | Art/ Klassifikation | Clav.Dindo Grad | Datum und Name Eintragender |
| --- | --- | --- | --- | --- |
|  |  |  |  |  |
|  |  |  |  |  |
|  |  |  |  |  |
|  |  |  |  |  |
|  |  |  |  |  |
|  |  |  |  |  |
|  |  |  |  |  |
|  |  |  |  |  |
|  |  |  |  |  |
|  |  |  |  |  |
|  |  |  |  |  |

**Komplikationen nach POD 30:** (früher aufgetretene Komplikationen bitte beim Komplikationsteil „Komplikationen bis inkl. POD 30“ eintragen)

Art der Komplikation: ⬜ ja ⬜ nein

Zeitpunkt des Auftretens: POD _____ oder Datum: ____/____/________

Wenn ja, Begründung angeben (inkl. postop. Tag, Intervention)

_________________________________________________________________

_________________________________________________________________

_________________________________________________________________

_________________________________________________________________

Endgültige Beurteilung:

Nach Clavien Dindo: I O II O IIIa O IIIb O IVa O IVb O V O

Komplikation: ⬜ ja ⬜ nein

Zeitpunkt des Auftretens: POD _____ oder Datum: ____/____/________

Wenn ja, Begründung angeben (inkl. postop. Tag, Intervention)

_________________________________________________________________

_________________________________________________________________

_________________________________________________________________

_________________________________________________________________

Endgültige Beurteilung:

Nach Clavien Dindo: I O II O IIIa O IIIb O IVa O IVb O V O

Zeitpunkt des Auftretens weiterer Komplikationen: Datum: ____/____/________

Bitte angeben: (Art der Komplikation, Begründung, Intervention, Re – Operation,

stationäre Wiederaufnahme etc.)

________________________________________________________________________

________________________________________________________________________

________________________________________________________________________

________________________________________________________________________

________________________________________________________________________

Zeitpunkt des Auftretens weiterer Komplikationen: Datum: ____/____/________

Bitte angeben: (Art der Komplikation, Begründung, Intervention, Re – Operation,

stationäre Wiederaufnahme etc.)

________________________________________________________________________

________________________________________________________________________

________________________________________________________________________

________________________________________________________________________

________________________________________________________________________

Interventionen nach Komplikationen: (bitte weitere nicht-erfasste Interventionen angeben)

Interventionen ⬜ ja ⬜ nein

Falls „ja“ bitte angeben:

Zeitpunkt: POD _____ oder Datum: ____/____/________

(Grund, Art)

_____________________________________________________________

_____________________________________________________________

_____________________________________________________________

_____________________________________________________________

Antibiotikatherapie ⬜ ja ⬜ nein

Falls „ja“ bitte angeben:

Zeitpunkt: POD _____ oder Datum: ____/____/________

(Grund, Dauer, Art)

_____________________________________________________________

_____________________________________________________________

_____________________________________________________________

_____________________________________________________________

Andere: ⬜ ja ⬜ nein

Falls „ja“ bitte angeben:

Zeitpunkt: Datum: ____/____/________

(Beschreibung, Grund)

_____________________________________________________________

_____________________________________________________________

_____________________________________________________________

_____________________________________________________________

Andere: ⬜ ja ⬜ nein

Falls „ja“ bitte angeben:

Zeitpunkt: Datum: ____/____/________

(Beschreibung, Grund)

_____________________________________________________________

_____________________________________________________________

_____________________________________________________________

_____________________________________________________________

| Ich bestätige nach bestem Wissen und Gewissen, dass die obigen Angaben vollständig, zutreffend und korrekt sind. | |
| --- | --- |
|  |  |
| Name des Prüfarztes | Unterschrift |

SF-36: (siehe vorgefertigter Ausdruck)

EORTC QLQ-C30


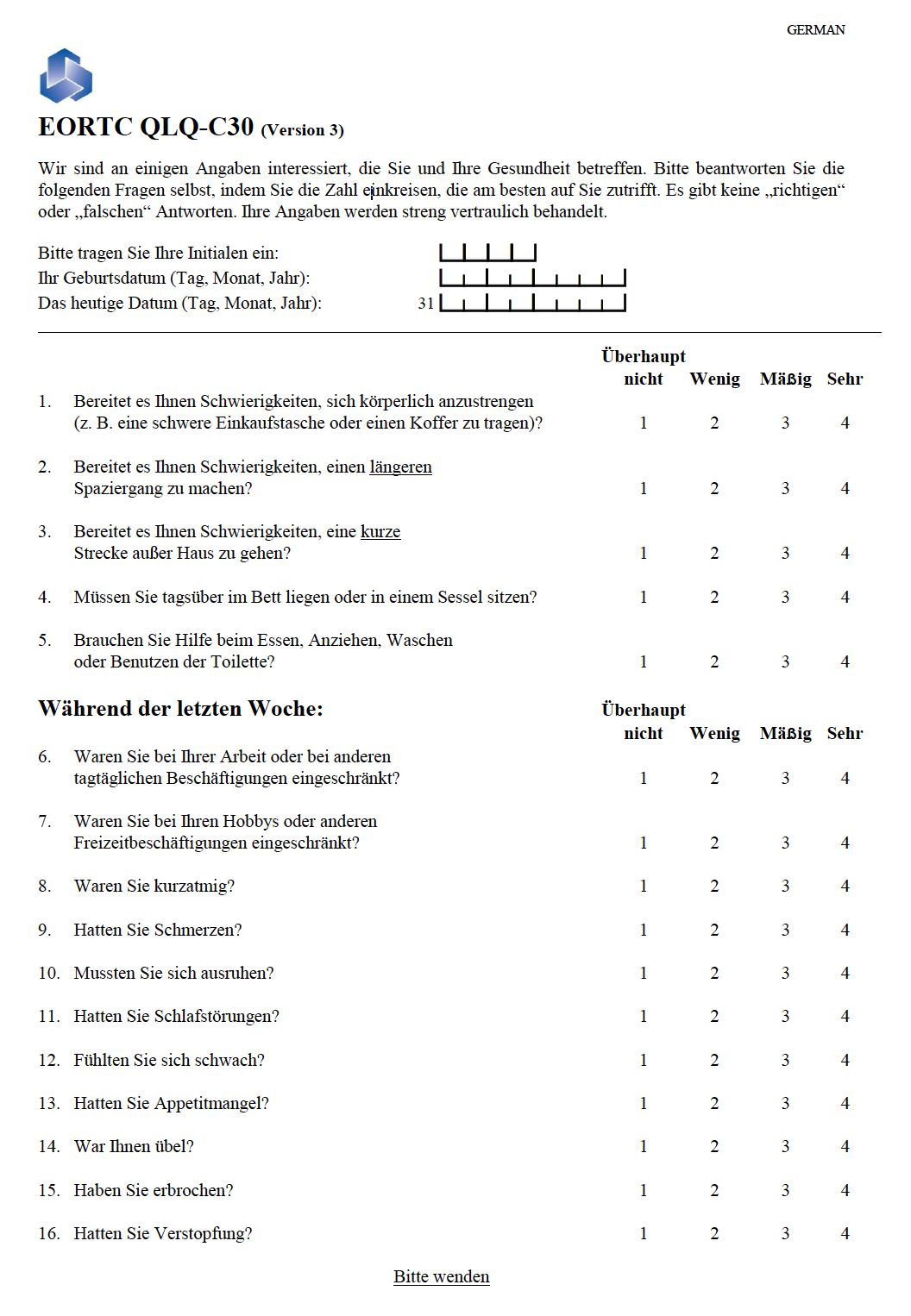


**
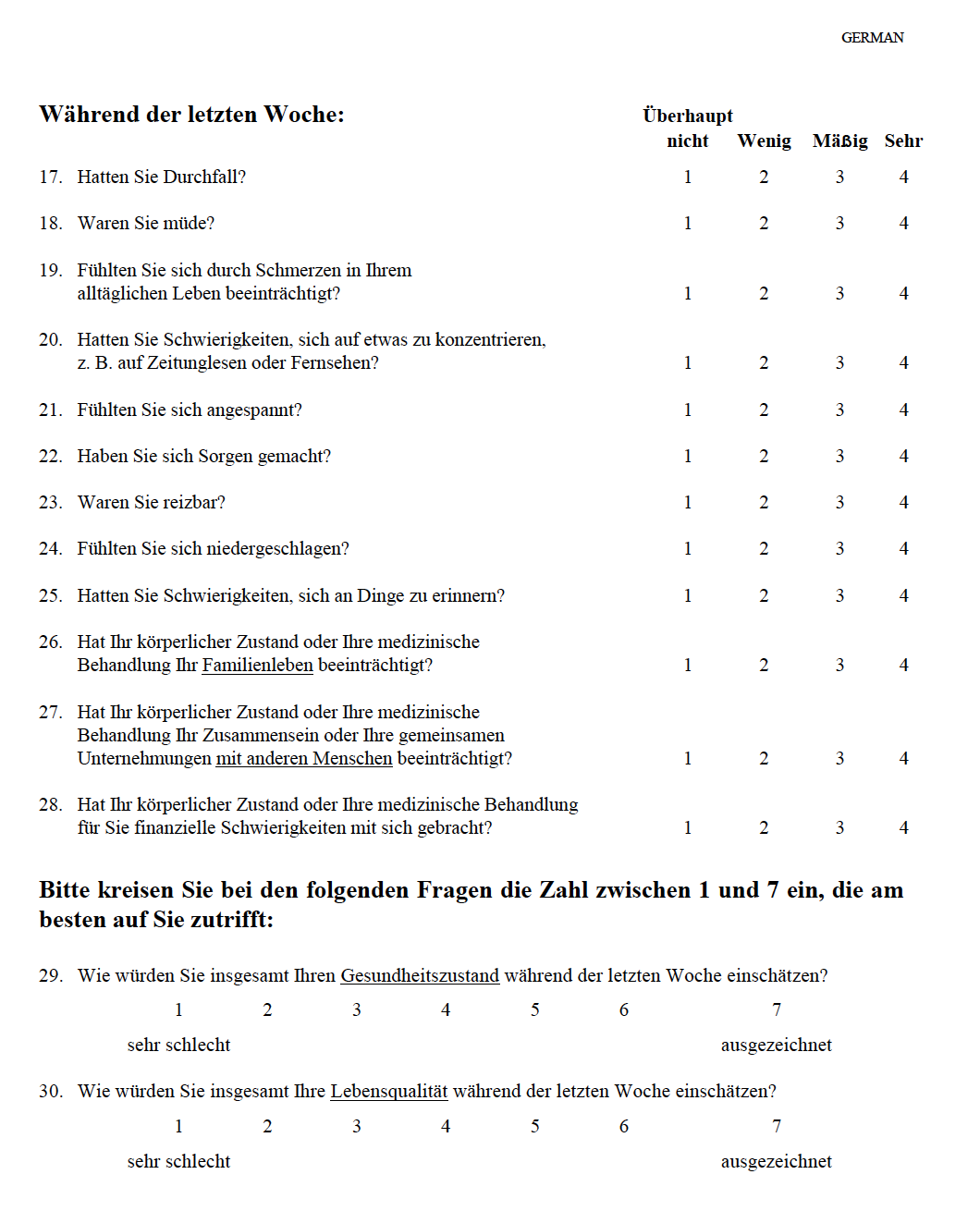
**

Summe: Datum:

EORTC QLQ-OES18

**
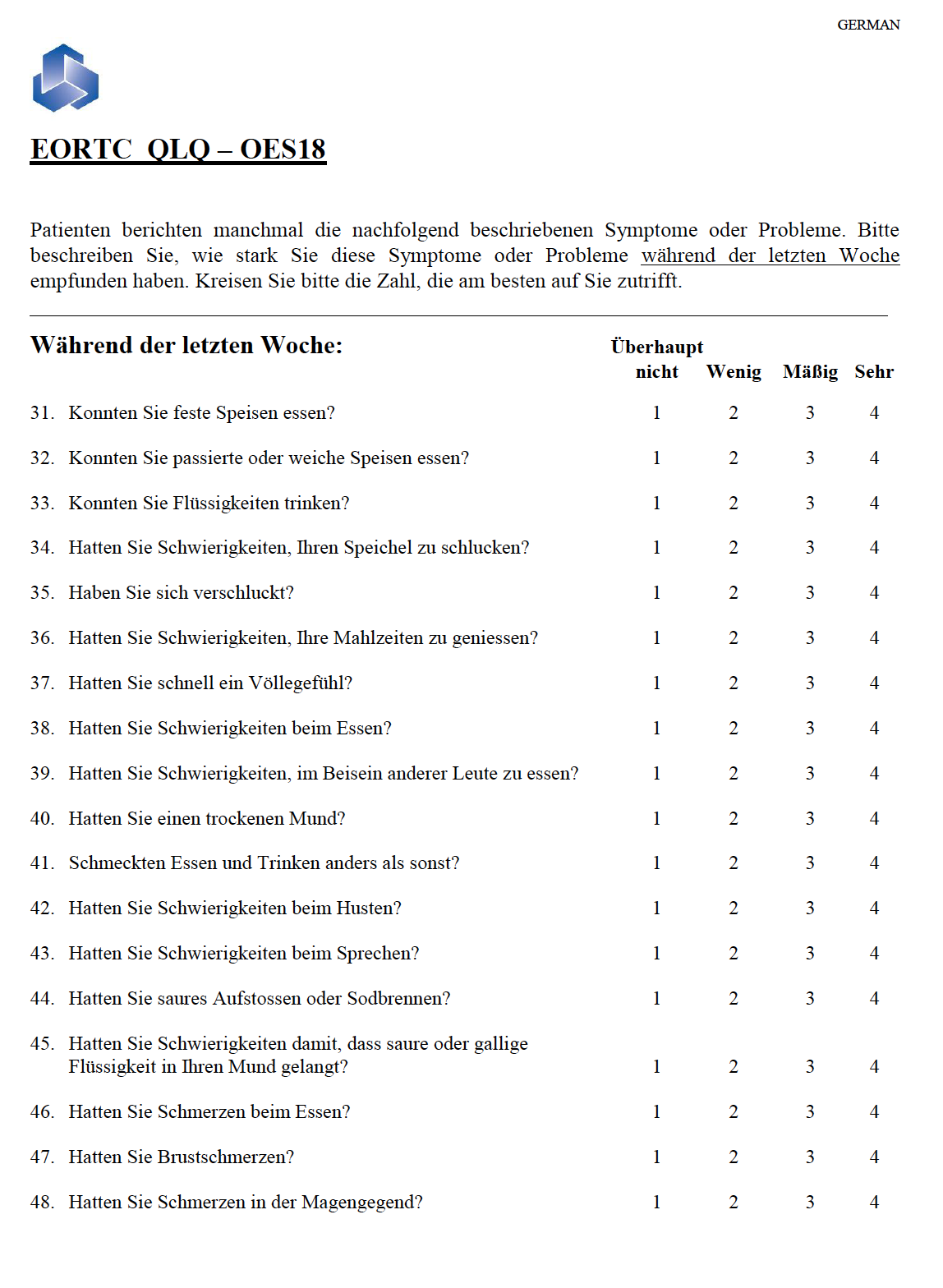
**

Summe: Datum:

QoR-15; Quality of Recovery 15

**
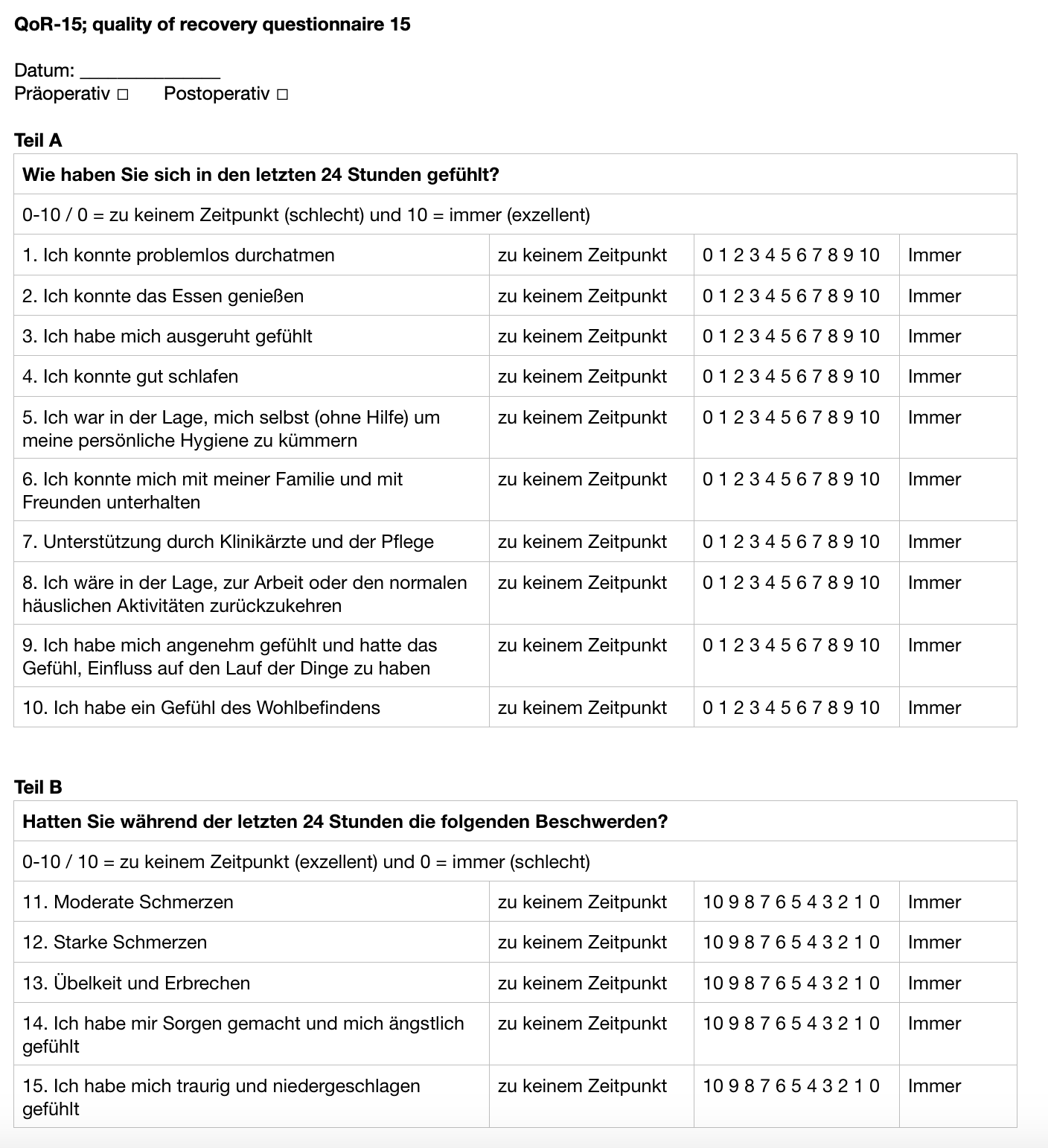
**

Summe Teil A: Summe Teil B:

Summe Gesamt: Datum:

**3. Follow-up (12 Monate) Datum: ____/____/__________**

⬜ Ja ⬜ Nein 🡪 Grund angeben:

⬜ Rücktritt des Patienten von der Studie ⬜ Patient verstorben

Datum: ___/___/______

⬜ Patient nicht mehr erreichbar ⬜ Anderer Grund: _________________

**Gewicht** [kg]: ____________

**Symptomerfassung:**

Reflux: ⬜ Ja ⬜ Nein Medikation: _____________________________

Dysphagie: ⬜ Ja ⬜ Nein Medikation: _____________________________

**Fragebögen:**

Lebensqualitätserfassung (SF-36) ⬜ _______________

EORTC QLQ-C30 ⬜ _______________

EORTC QLQ- OES18 ⬜ _______________

QoR – 15: ⬜ _______________

Rückkehr zur Arbeit/ normalen (häuslichen) Aktivitäten: ____/____/_______

**Lokalrezidiv oder Progressive Disease**:

⬜ Ja ⬜ Nein, Beschreibung: _________________________________________

**Adjuvante Therapie**:

Wirkstoff (Dosis)__________________________ Zyklen: ____________________

Wirkstoff (Dosis)__________________________ Zyklen: ____________________

Wirkstoff (Dosis)__________________________ Zyklen: ____________________

**Morbidität/ Mortalität (> 30 Tage)**

⬜ nein ⬜ ja 🡪 weiter mit „Komplikationen Erfassung nach POD 30“

## **Name und Unterschrift des Prüfers**:

**4. Follow-up (36 Monate) Datum: ____/____/__________**

⬜ Ja ⬜ Nein 🡪 Grund angeben: _____/_____/_____________

⬜ Rücktritt des Patienten von der Studie ⬜ Patient verstorben

Datum: ___/___/______

⬜ Patient nicht mehr erreichbar ⬜ Anderer Grund: _________________

**Gewicht** [kg]: ____________

**Symptomerfassung:**

Reflux: ⬜ Ja ⬜ Nein Medikation: _____________________________

Dysphagie: ⬜ Ja ⬜ Nein Medikation: _____________________________

**Fragebögen:**

Lebensqualitätserfassung (SF-36) ⬜ _______________

EORTC QLQ-C30 ⬜ _______________

EORTC QLQ- OES18 ⬜ _______________

QoR – 15: ⬜ _______________

Rückkehr zur Arbeit/ normalen (häuslichen) Aktivitäten: ____/____/______

**Lokalrezidiv oder Progressive Disease**:

⬜ Ja ⬜ Nein, Beschreibung: _________________________________________

**Adjuvante Therapie**:

Wirkstoff (Dosis)__________________________ Zyklen: ____________________

Wirkstoff (Dosis)__________________________ Zyklen: ____________________

Wirkstoff (Dosis)__________________________ Zyklen: ____________________

**Morbidität/ Mortalität (> 30 Tage)**

⬜ nein ⬜ ja 🡪 weiter mit „Komplikationen Erfassung nach POD 30“

## **Name und Unterschrift des Prüfers**:

**4. Follow-up (60 Monate) Datum: ____/____/__________**

⬜ Ja ⬜ Nein 🡪 Grund angeben: _____/_____/_____________

⬜ Rücktritt des Patienten von der Studie ⬜ Patient verstorben

Datum: ___/___/______

⬜ Patient nicht mehr erreichbar ⬜ Anderer Grund: _________________

**Gewicht** [kg]: ____________

**Symptomerfassung:**

Reflux: ⬜ Ja ⬜ Nein Medikation: _____________________________

Dysphagie: ⬜ Ja ⬜ Nein Medikation: _____________________________

**Fragebögen:**

Lebensqualitätserfassung (SF-36) ⬜ _______________

EORTC QLQ-C30 ⬜ _______________

EORTC QLQ- OES18 ⬜ _______________

QoR – 15: ⬜ _______________

Rückkehr zur Arbeit/ normalen (häuslichen) Aktivitäten: ____/____/______

**Lokalrezidiv oder Progressive Disease**:

⬜ Ja ⬜ Nein, Beschreibung: _________________________________________

**Adjuvante Therapie**:

Wirkstoff (Dosis)__________________________ Zyklen: ____________________

Wirkstoff (Dosis)__________________________ Zyklen: ____________________

Wirkstoff (Dosis)__________________________ Zyklen: ____________________

**Morbidität/ Mortalität (> 30 Tage)**

⬜ nein ⬜ ja 🡪 weiter mit „Komplikationen Erfassung nach POD 30“

(Zum Studienende weiter mit nächster Seite)

| **Studienende** | | | |
| --- | --- | --- | --- |
| Hat der Patient die Studie regulär beendet? | | | ja O nein O |
| Falls nein, bitte Grund für vorzeitiges Studienende angeben | | | |
| 1. Patient verstorben | | ja O nein O | |
| Wenn ja, Todesdatum | |  | |
| Wenn ja, Todesursache | _______________________________________________________________________________________________________ | | |
| 2. Lost to follow up | | ja O nein O | |
| Wenn ja, Datum des letzten Kontaktes | |  | |
| 3. Widerruf der Einwilligungserklärung | | ja O nein O | |
| Wenn ja, Datum des Widerrufs | |  | |
| 4. anderer | | ja O | |
| Wenn ja, bitte angeben | ________________________________________________________________________________________________________________ | | |
| __________________________________________________________________________________________________________________________________________________________________________________ | | | |

## **Name und Unterschrift des Prüfers**:

# Teil 2: Fragebögen

**Anhang Teil 1**

1. QoR-15 (Quality of Recovery 15)

2. SF-36 Fragebogen

3. EORTC QLQ-C30

4. EORTC QLQ-OES18

5.1 Clavien – Dindo – Klassifikation

5.2 Comprehensive Complication Index (CCI)

6. CDC – Definition von Wundinfektionen

7. ASEPSIS Score

**Teil 3: Definitionen: Anhang Teil 2**

8. SOP – Leitfaden für das postoperative Vorgehen nach Ösophagektomie

9. Überblick über die postoperativen Komplikationen

1. QoR-15; Quality of Recovery 15

**
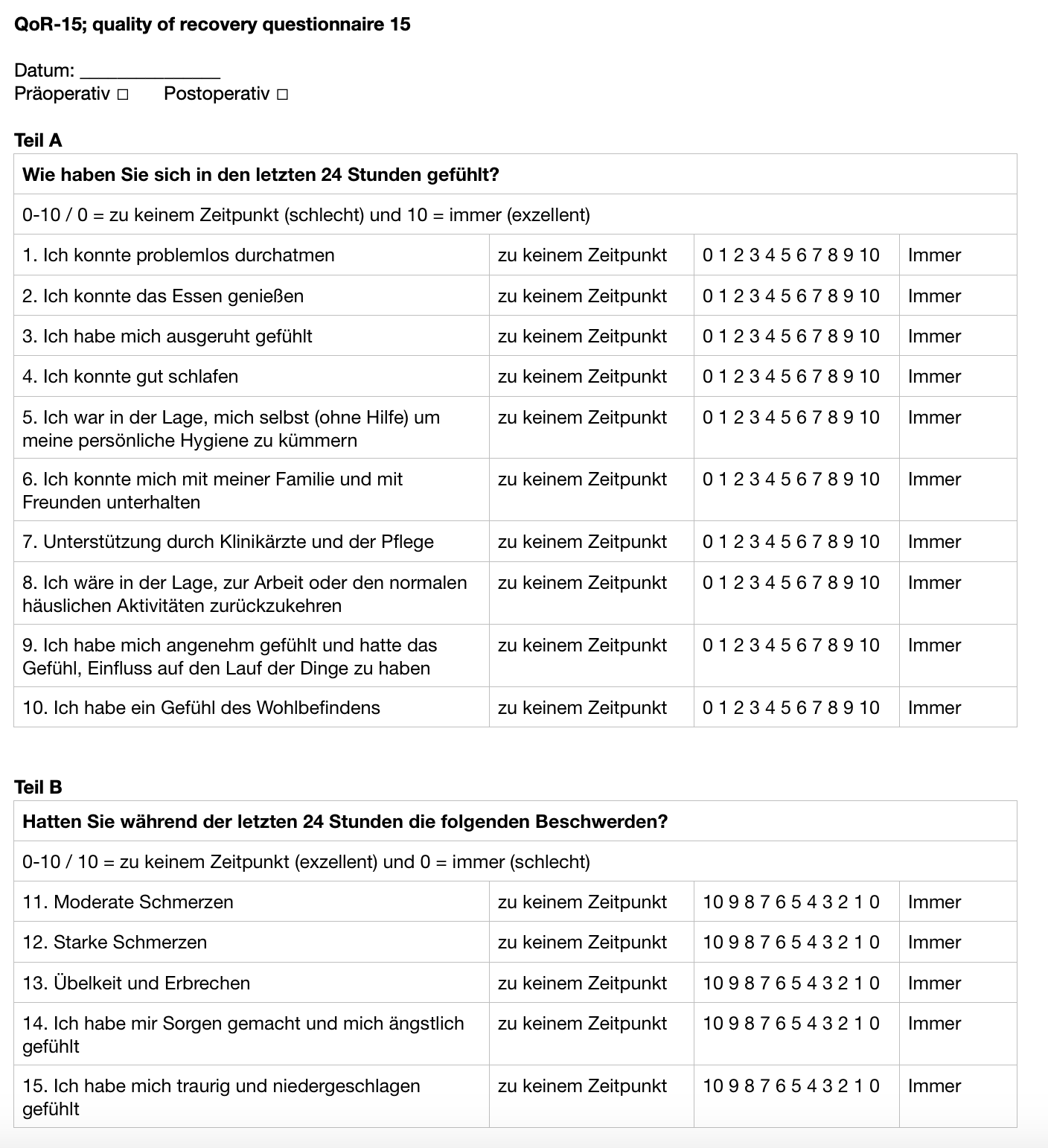
**

Summe Teil A: Summe Teil B:

Summe Gesamt: Datum:

2. SF-36 (Siehe Ausdruck)


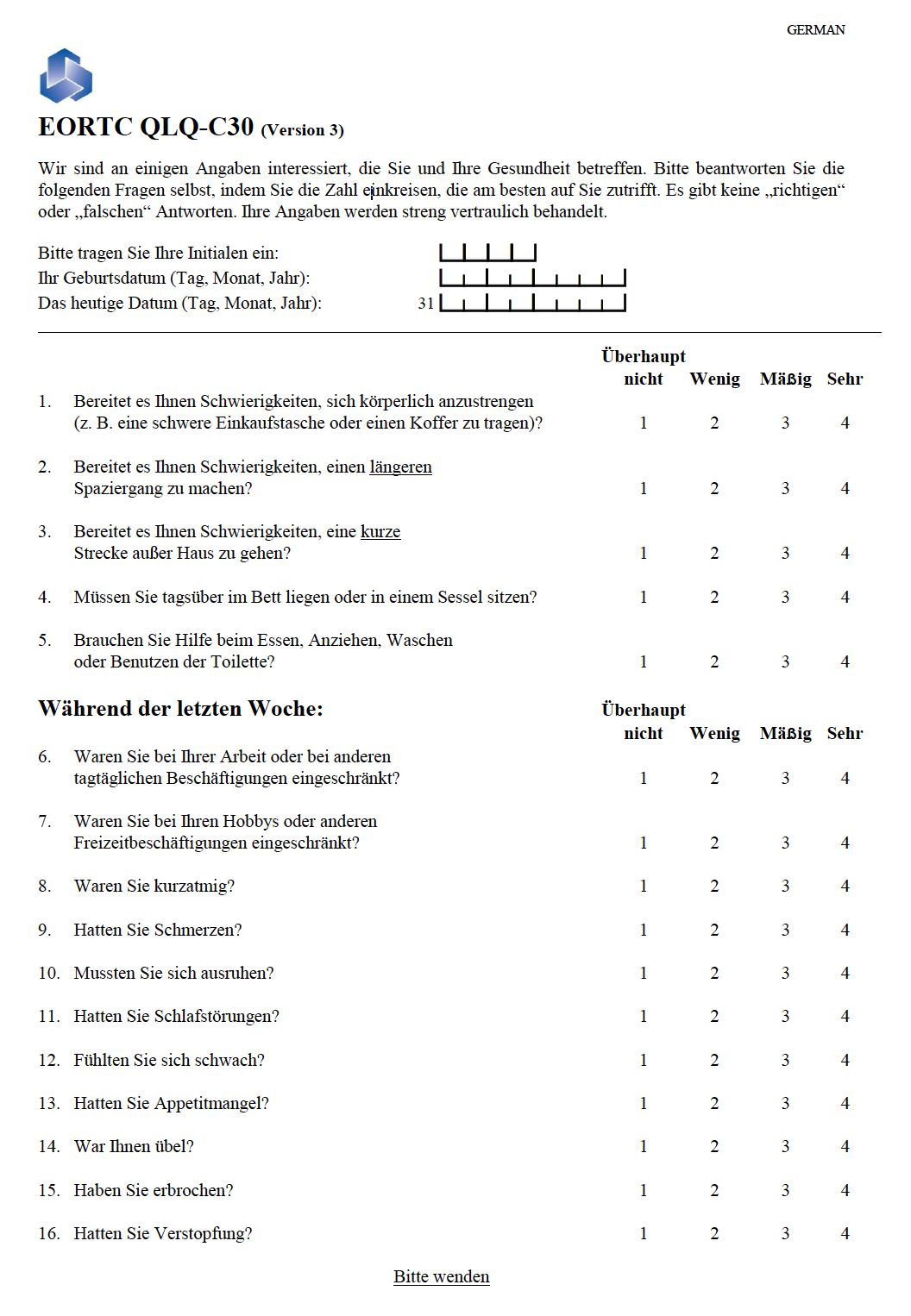
3. EORTC QLQ-C30

**
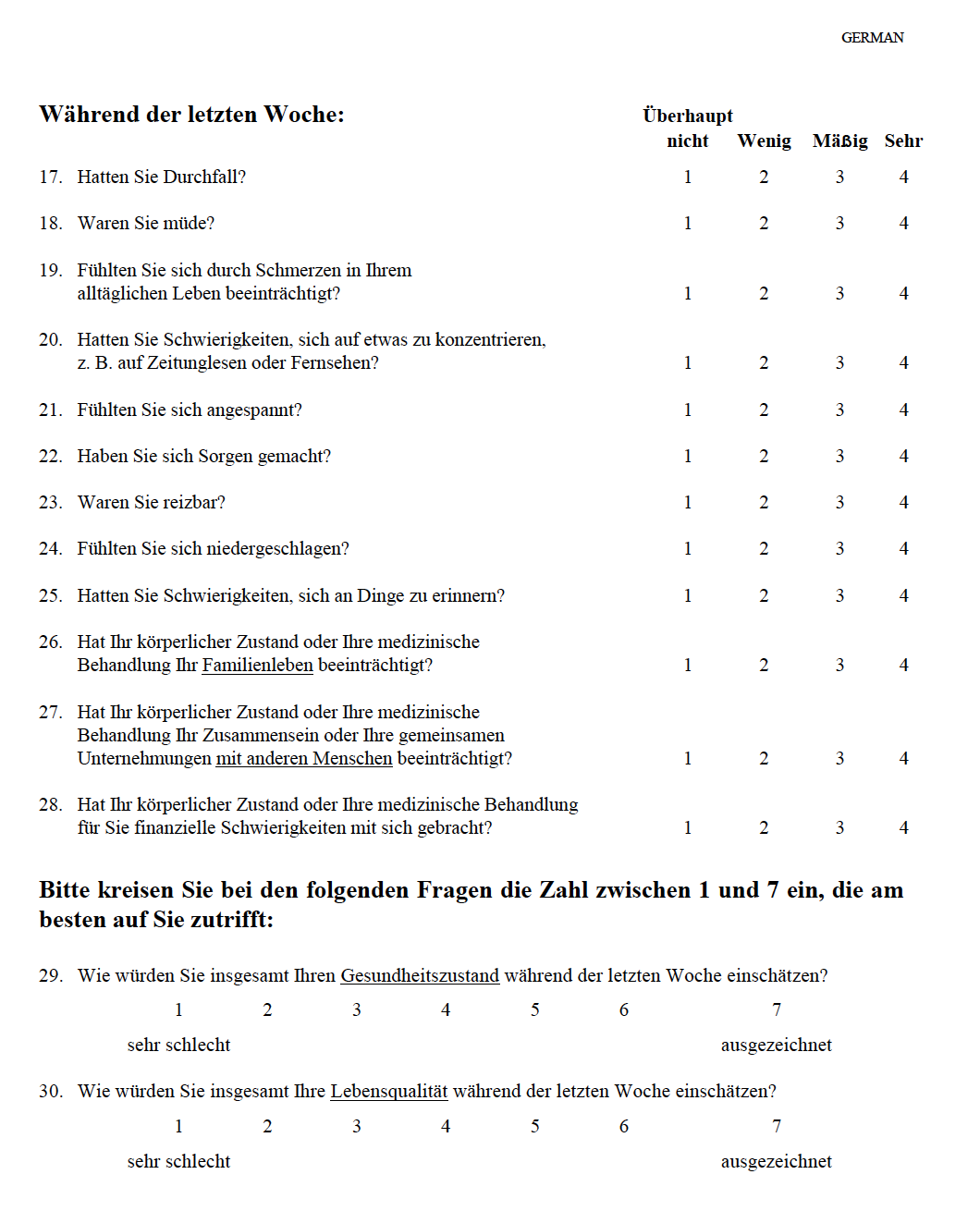
**

Summe: Datum:

4. EORTC QLQ-OES18

**
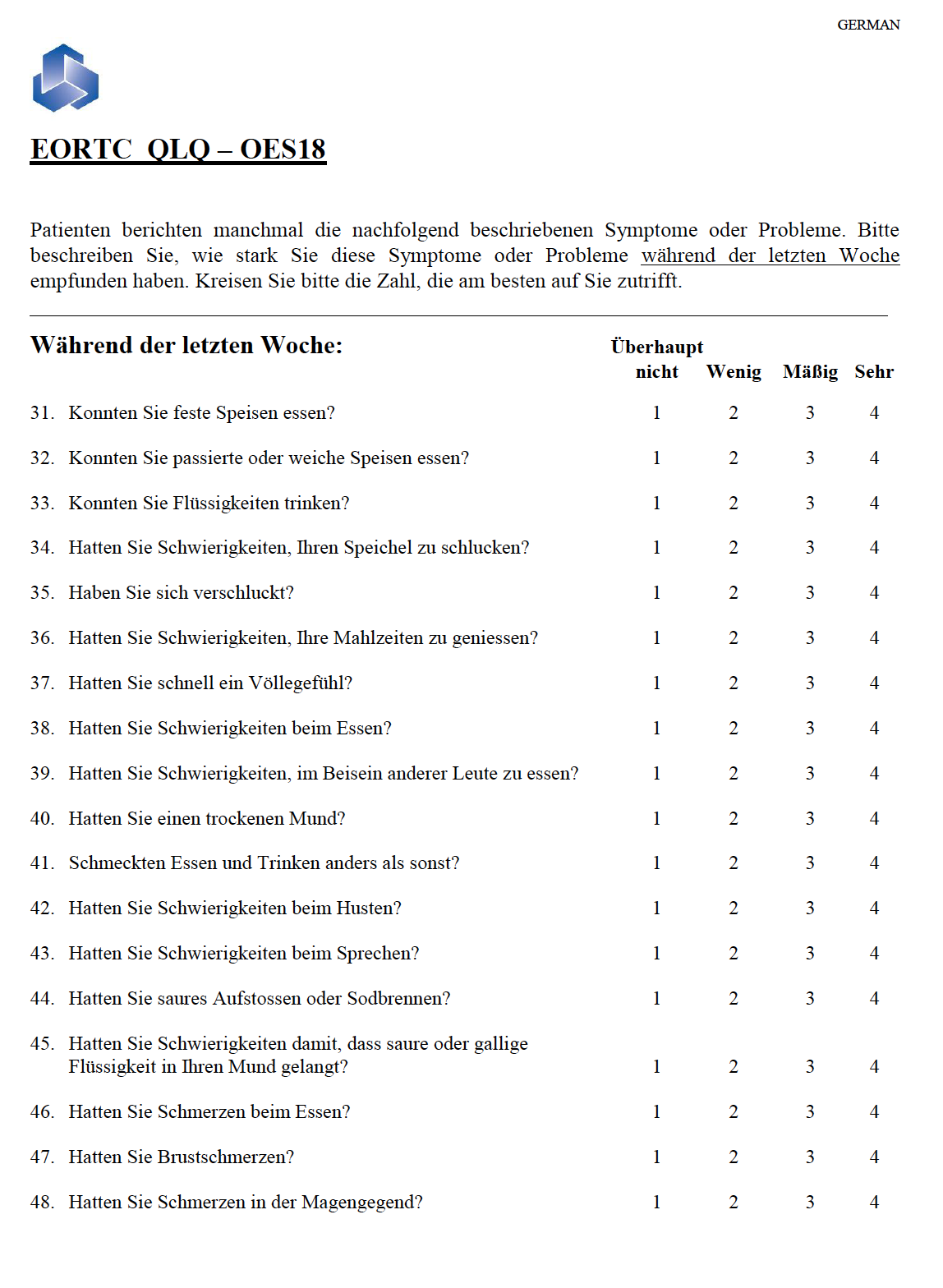
**

Summe: Datum:

**5. Comprehensive Complication Index (CCI®)**

5.1 Clavien – Dindo – Klassifikation:

|  | Definition |
| --- | --- |
| ⬜ Grad I | Jede Abweichung vom normalen stationären Verlauf aber ohne, dass eine spezifische medikamentöse oder chirurgische Behandlung notwendig war. Ausgeschlossen sind radiologische oder endoskopische Intervention. Erlaubte medikamentöse Therapien sind: Antiemetische, antipyretische, analgetische und diuretische Therapien sowie Elektrolyttherapie. Physiotherapie ist gestattet. Oberflächliche Wundinfektionen, welche mit bettseitigem Eröffnen der Wunde einhergehen sind ebenfalls gestattet. |
| ⬜ Grad II | Medikamentöse Therapie, die über die erlaubten aus der Grad I hinaus gehen. Bluttransfusion und parenterale Ernährung gehören ebenfalls hinzu. |
| ⬜ Grad III | Chirurgische, endoskopische oder radiologische Interventionen erforderlich |
| ⬜ Grad IIIA | ohne Allgemeinanästhesie |
| ⬜ Grad IIIB | mit Allgemeinanästhesie |
| ⬜ Grad IV | Lebensbedrohliche Komplikation, die eine Behandlung auf Intensivstation notwendig werden lässt |
| ⬜ Grad IVA | Einzelnes Organversagen (inkl. Dialyse) |
| ⬜ Grad IV B | Multiorganversagen |
| ⬜ Grad V | Tod des Patienten |

[**https://www.assessurgery.com/about_cci-calculator/**](https://www.assessurgery.com/about_cci-calculator/)

5.2 CCI – Calculator:

**
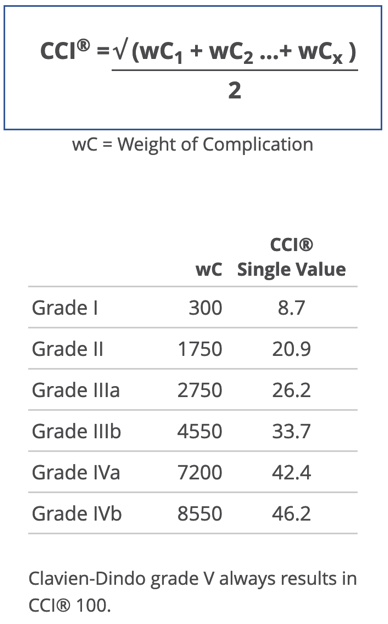
**

Erreichter Grad laut CDK:

Summe laut CCI:

Datum:

6. Wundheilungsstörung: Definition der Wundinfektion nach CDC (Center for Disease Control & Prevention)

**Superficial** incisional SSI:

Infection occurs within 30 days after an operative procedure, and involves only skin and subcutaneous tissue of the incision and patient has at least one of the following:

1. Purulent draining from the superficial incision.
2. Organisms isolated from an aseptically obtained culture of fluid or tissue from the superficial incision (incisional drainage).
3. At least one of the following signs or symptoms of infection: pain or tenderness, localized swelling, redness or heat, and superficial incision is deliberately opened by surgeon, and is culture positive or not cultured.
4. Diagnosis of superficial incisional SSI by the surgeon or attending physician.

**Deep** incisional SSI:

Infection occurs within 30 or 90 days after the operative procedure and involves deep soft tissues (fascial and muscle layers) of the incision and patient has at least one of the following:

1. Purulent drainage from the deep incision but not from the organ/space component of the surgical site.
2. A deep incision spontaneously dehisces or is deliberately opened by a surgeon and is culture positive or not cultured and when the patient has at least one of the following signs or symptoms: fever (>38°C), localized pain, or tenderness, unless site is culture negative.
3. An abscess or other evidence of infection involving the deep incision is found on direct examination, during reoperation, or by histopathologic or radiologic examination.
4. Diagnosis of a deep incisional SSI by a surgeon or attending physician.

**Organ/Space** SSI:

Infection occurs within 30 or 90 days after the operation and infection involves any part of the anatomy (organs or spaces), other than the skin incision, fascia or muscle layers that is opened or manipulated during an operation and at least one of the following:

1. Purulent drainage from a drain that is placed into the organ/space.
2. Organisms isolated from an aseptically obtained culture or fluid or tissue in the organ/space.
3. An abscess or other evidence of infection involving the organ/space that is found on direct examination, during reoperation, or by histopathologic or radiologic examination.
4. Diagnosis of an organ/space SSI by a surgeon or attending physician.

7. ASEPSIS Score (bitte Zutreffendes markieren)


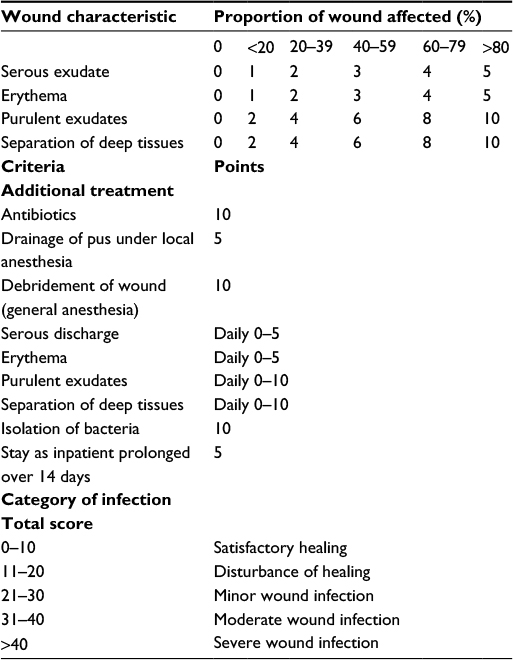


Wilson AP, Treasure T, Sturridge MF, Gruneberg RN. A scoring method (ASEPSIS)

for postoperative wound infections for use in clinical trials of antibiotic prophylaxis.

*Lancet* 1986; 1: 311-13.

# Teil 3: Definitionen

8. SOP – Leitfaden für das postoperative Vorgehen nach Ösophagektomie


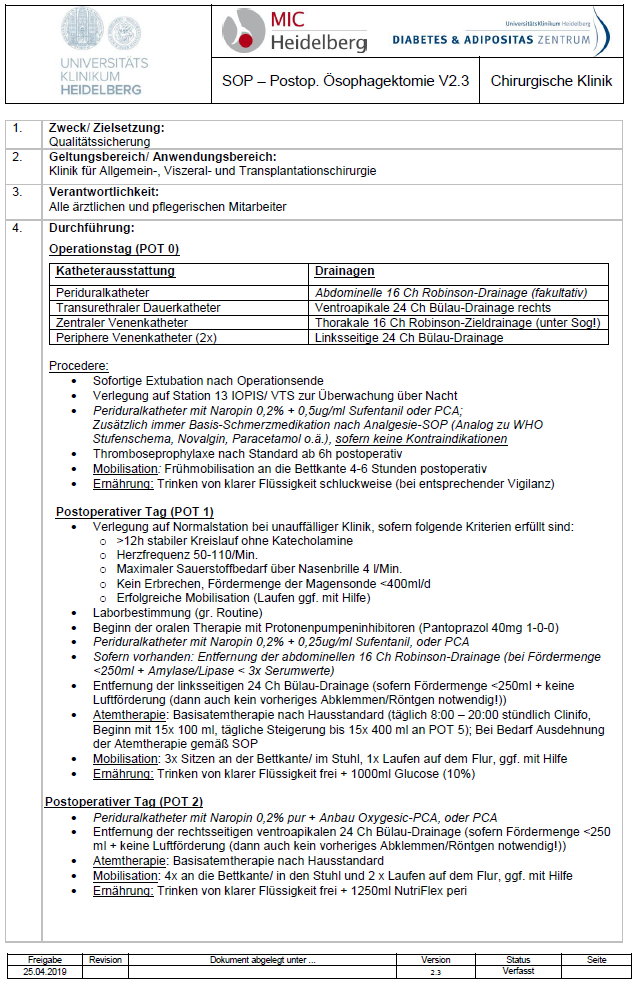


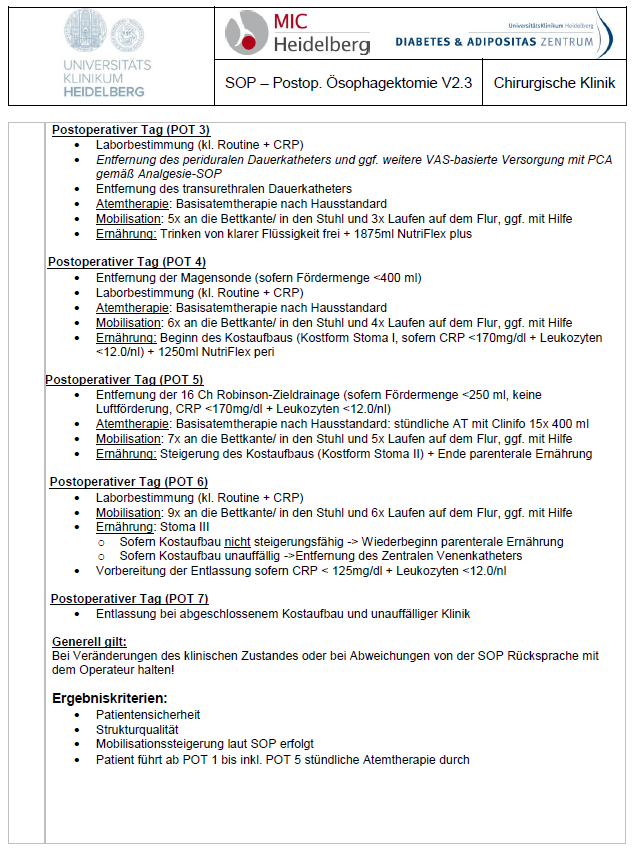


9. Überblick über postoperative Komplikationen

ECCG – Definitionen:


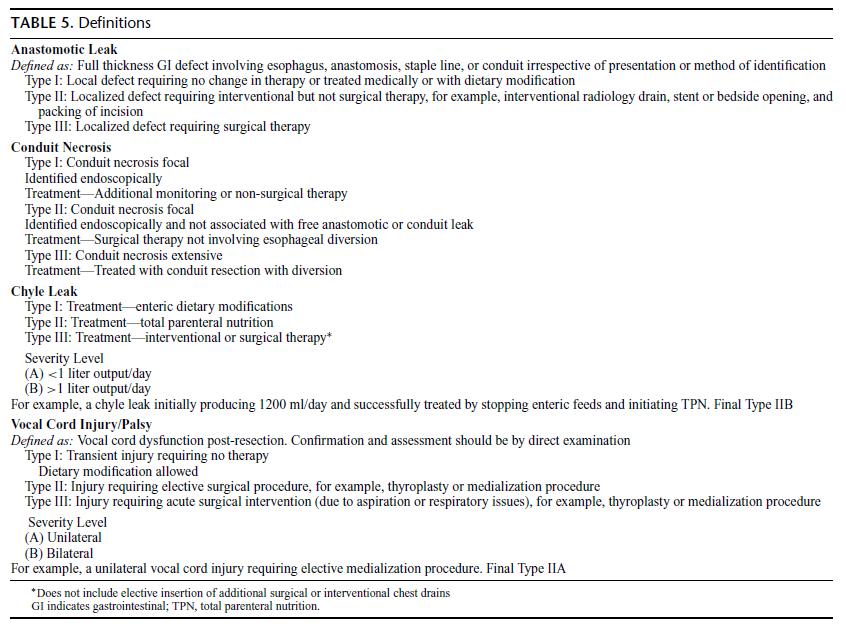


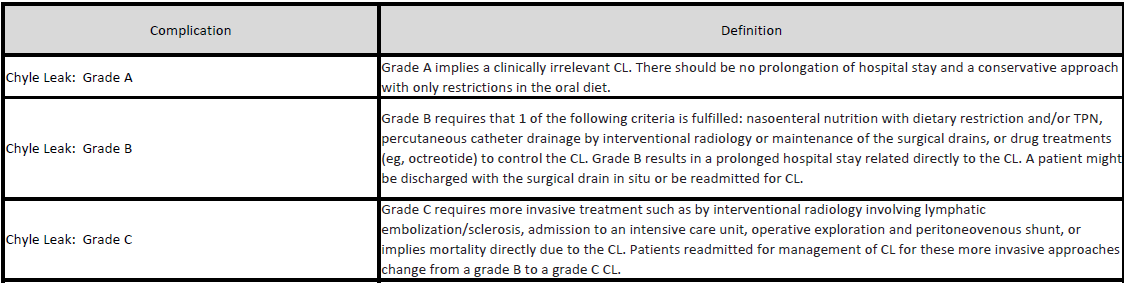


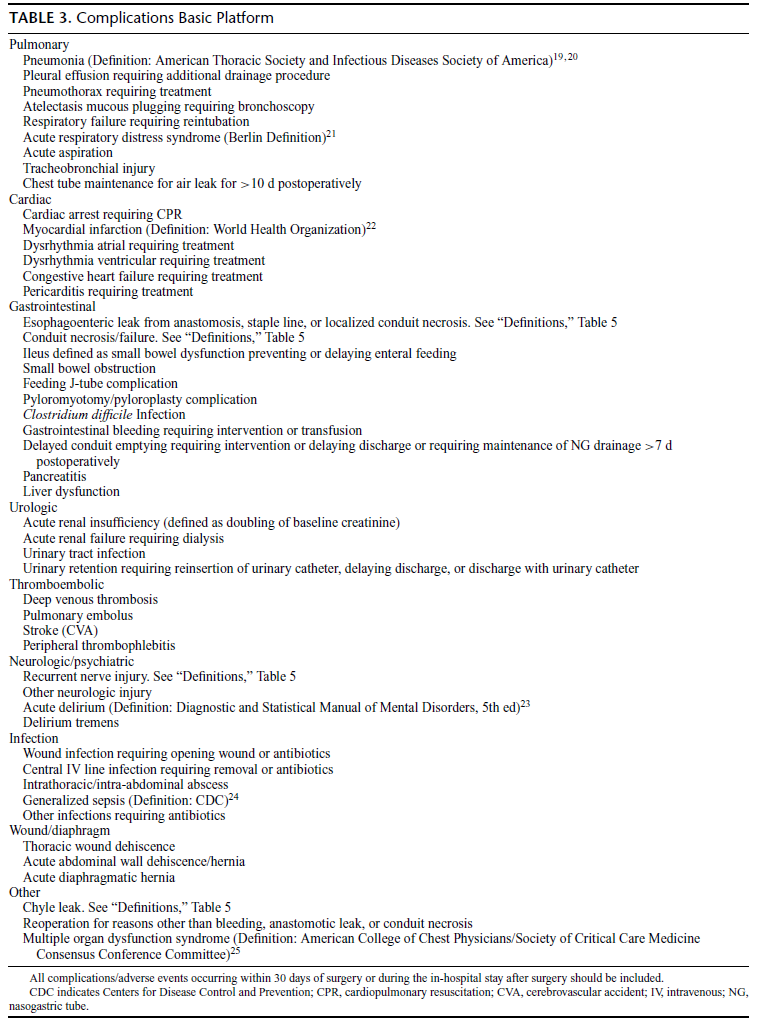


D.E. Low et.al. – Internat. Consensus on Standardization of Data Collection for Complications Associated With Esophagektomie. Ann Surg 2015; 262:286-294

1. Stomakost 1 – 3 zum stufenweisen Kostaufbau

   **1 =** Energiegehalt 500 kcal/ 2093 KJ; Bestehend aus Haferschleim, fettarme Brühe/ Cremesuppen,

   Zwieback, Götterspeise. Keine Rohkost außer Banane. 5 Portionen pro Tag. Nährstoffrelation 15%

   Eiweiß, 29% Fett, 56% Kh.

   Überleitung zur Stufe 2: Kartoffelpüree & braune Soße, Nudeln & milde Tomatensauce, Grießbrei, Reisbrei, Banane und fettarme Joghurt / Buttermilch.

   **2 =** Energiegehalt 1700 kcal/ 7116 KJ; Gut bekämmliche Getreideerzeugnisse, leicht verdauliche,

   fettreduzierte tierische Eiweßträger (Käse, Kuhmilch, Wurst). Leicht verdauliche Koch – und

   Streichfette ansonsten Produkte mit max. 3,5% Fettgehalt. Keine Rohkost außer Banane. 6

   Portionen pro Tag. 12% Eiweiß, 30% Fett, 58% Kh.

   **3 =** E-Gehalt 1800 kcal/ 3348 KJ. Erweiterung des Mittagessens durch bindegewebsarmes Fleisch. Kleine

   Mengen Rohkost mgl. Keine Pflanzenfasern wie Spargel / Organe. Bevorzugung von

   Stuhleindickenden Lebensmitteln. 6 Portionen pro Tag. 15% Eiweiß, 30-35% Fett, 50-55% Kh. [↑](#footnote-ref-1)
